# RNA-binding landscape of amiloride: large-scale profiling and structural basis of U–U mismatch recognition

**DOI:** 10.1039/d6cb00055j

**Published:** 2026-04-15

**Authors:** Kosuke Tsuzuki, Kazumitsu Onizuka, Momo Okada, Ryosuke Nagasawa, Emi Miyashita, Kaoru R. Komatsu, Hirohide Saito, Jiro Kondo, Fumi Nagatsugi

**Affiliations:** a Institute of Multidisciplinary Research for Advanced Materials, Tohoku University Miyagi 980-8577 Japan onizuka@tohoku.ac.jp nagatugi@tohoku.ac.jp; b Department of Chemistry, Graduate School of Science, Tohoku University Miyagi 980-8578 Japan; c Division for the Establishment of Frontier Sciences of Organization for Advanced Studies, Tohoku University Miyagi 980-8577 Japan; d Department of Materials and Life Sciences, Faculty of Science and Technology, Sophia University Tokyo 102-8554 Japan j.kondo@sophia.ac.jp; e Center for iPS Cell Research and Application (CiRA), Kyoto University Kyoto 606-8507 Japan; f Institute for Quantitative Biosciences, The University of Tokyo Tokyo 113-0032 Japan

## Abstract

Amiloride possesses a characteristic chemical scaffold capable of recognizing uracil (U) through three complementary hydrogen bonds; however, its binding selectivity toward naturally occurring RNA structural motifs has remained uncharacterized. In this study, we present a large-scale analysis of amiloride's RNA binding properties and structural characterization of the amiloride–RNA complex. Using folded RNA element profiling with structure library (FOREST), we evaluated the RNA-binding selectivity of amiloride across 3000 structured RNA motifs and uncovered pronounced binding preferences for G-quadruplexes and, notably, for a specific internal loop motif containing a U–U mismatch (*K*_Dapp_ = 0.31 µM). Furthermore, a motif extraction strategy was used to enable detailed structural investigation. The X-ray crystal structure of the amiloride–RNA complex provides the first structural evidence that amiloride recognizes a U residue within a naturally occurring RNA context *via* its signature complementary hydrogen bonding interactions.

## Abstract 2

Comprehensive FOREST profiling identified a U–U mismatch RNA motif to which amiloride strongly binds. X-ray crystallography revealed the precise binding mode, driven by three complementary hydrogen bonds with uracil and stacking interactions.

## Graphical abstract

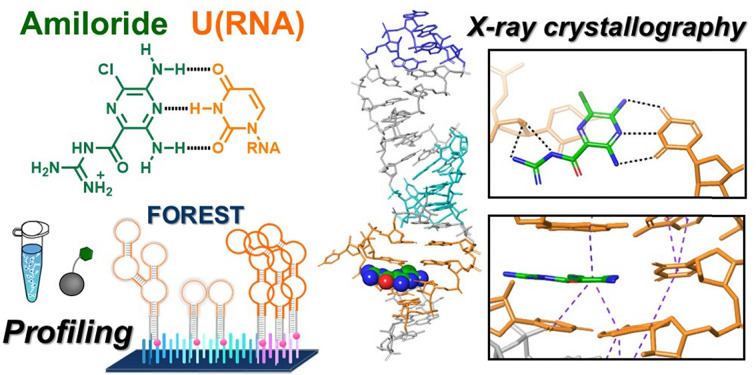

## Introduction

**Fig. 1:**
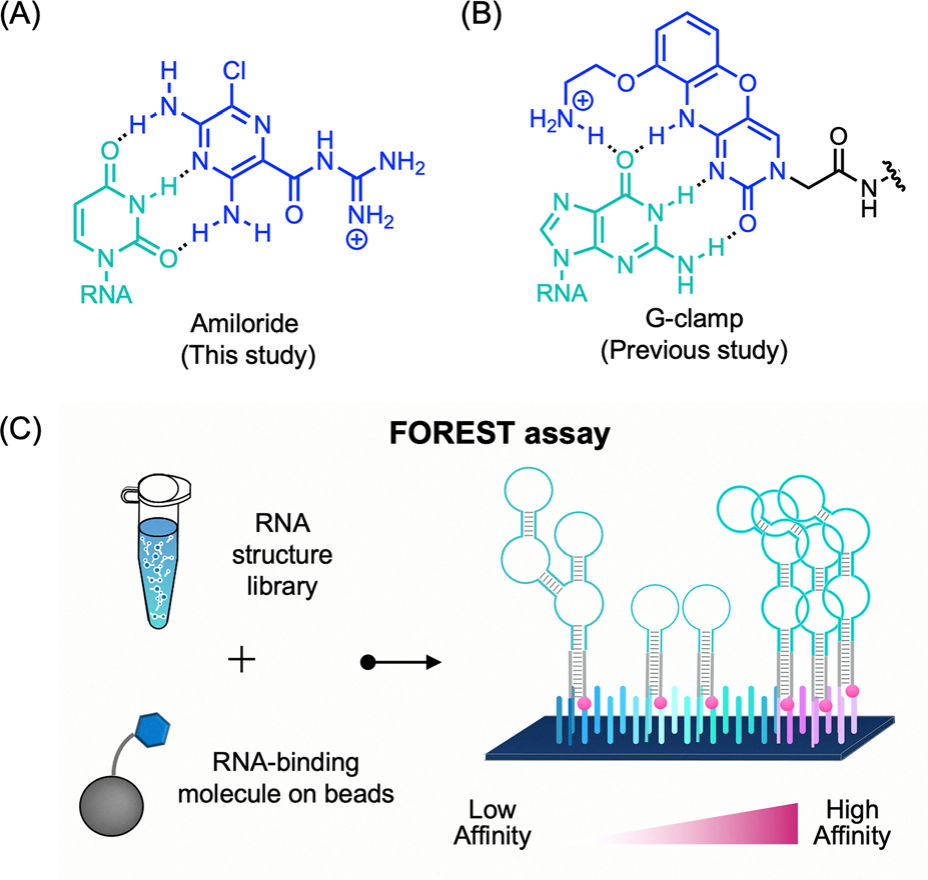
Schematic overview of this study. (A) Predicted hydrogen-bonding interaction between uracil and amiloride. (B) Known recognition mode of guanine by G-clamp. (C) Conceptual summary of the FOREST assay for large-scale profiling of the RNA-binding selectivity of small molecules.

Small molecules capable of recognizing specific nucleobases in DNA and RNA through complementary hydrogen-bond formation are powerful tools in chemical biology. Their application includes detection of nucleic acids,^1,2^ molecular glue,^3^ modulation of microRNA maturation,^4,5^ targeting pathogenic repeat expansion RNAs,^6,7^ and construction of nucleic acid-based architectures.^8,9^ Among such molecules, amiloride forms a pseudo-base pair with a uracil (U) (Fig. 1A). Although amiloride is known to recognize and bind tightly to unpaired U residues in an artificial cavity created by incorporating a C3 spacer in RNA duplexes,^10^ and its derivatives have shown broad binding activity toward various viral RNAs,^11–17^ its binding behavior in naturally occurring RNA structures remains largely unexplored. In particular, despite its potential for high-affinity U recognition, large-scale evaluations of its RNA-binding selectivity and direct experimental evidence of its characteristic triple hydrogen-bonding interactions with naturally occurring RNA targets have not yet been reported.

While addressing these questions requires a systematic analysis of ligand–RNA interactions, foundational efforts have already formalized statistical frameworks for extracting these relationships from RNA motif libraries, introducing motif-based screening and quantitative enrichment analyses, as well as comprehensive RNA-binding selectivity profiling.^18–20^ Building on these pioneering methodologies, we previously developed the FOREST (folded RNA element profiling with structure library) platform, which employs a structurally diverse library of naturally occurring human RNA sequences, encompassing internal loops, bulges, multi-loops, and other non-canonical motifs, for *Z*-score-based, large-scale profiling of small molecule-RNA interactions (Fig. 1 and Fig. S1).^21,22^ This system enables the unbiased identification of binding preferences across a broad structural landscape that extends beyond the combinatorial motif libraries used in prior enrichment-based approaches. In particular, the utility of this approach for characterizing hydrogen-bond-mediated interactions was previously demonstrated using G-clamp, a cytosine analog that strongly recognizes guanine (G) (Fig. 1B).^23^ In our previous work, large-scale binding profiling revealed that G-clamp does not bind promiscuously to all unpaired guanine residues but instead exhibits a strict preference for specific unpaired guanines in distinct structural contexts. Identification of such high-affinity motifs through comprehensive screening paved the way for the determination of the high-resolution X-ray crystal structure of the G-clamp-RNA complex, providing atomic-level insight into its binding mode.^24^ Furthermore, these data facilitated the development of thiazole orange (TO)-G-clamp conjugates as fluorogenic probes for fluorescence indicator displacement (FID) assays, thereby enabling the discovery of novel RNA-binding molecules.^25^

**Fig. 2:**
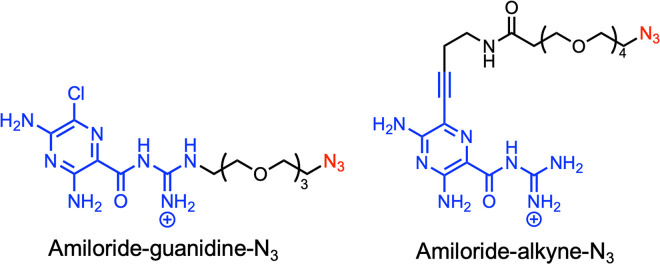
Molecular design of the amiloride derivatives (amiloride-guanidine-N_3_ and amiloride-alkyne-N_3_) used in this study.

In this study, we employed FOREST to systematically evaluate the RNA-binding properties of amiloride. First, we designed and synthesized amiloride derivatives for the FOREST assay while preserving the core hydrogen-bonding face of the molecule (Fig. 2). The resulting RNA-binding profiles were subsequently validated by fluorescence titration experiments, and the effects of the chemical modifications introduced for the FOREST analysis were further examined using surface plasmon resonance (SPR). Most notably, we identified a high-affinity interaction between amiloride and the internal loop structure of pre-miR-6074. Finally, X-ray crystallographic analysis of the amiloride–RNA complex provided, to our knowledge, the first atomic-resolution evidence that amiloride recognizes a U residue within a U–U mismatch in a naturally occurring RNA structure through its characteristic triple hydrogen-bonding pattern.

## Results and discussion

### Molecular design of amiloride derivatives

First, to enable immobilization on beads, two azide-modified amiloride derivatives with different linker attachment positions were designed. To preserve the hydrogen-bond donor–acceptor pattern of amiloride, the linker was introduced either at the guanidino group (amiloride-guanidine-N_3_) or at the pyrazine ring *via* an alkyne modification (amiloride-alkyne-N_3_). The designed amiloride derivatives were synthesized according to Schemes S1 and S2. Specifically, amiloride-guanidine-N_3_ was synthesized by conjugating a guanidinylated PEG linker to commercially available methyl 3,5-diamino-6-chloropyrazine-2-carboxylate. For the synthesis of amiloride-alkyne-N_3_, the chlorine of amiloride was first replaced with iodine, followed by Sonogashira coupling and subsequent amide condensation reactions. Prior to the FOREST assay, azide-modified amiloride derivatives were biotinylated *via* strain-promoted azide–alkyne cycloaddition (SPAAC) reaction with DBCO-SS-biotin. The formations of the biotinylated products were confirmed by RP-HPLC and ESI-HRMS (SI).

### RNA-binding selectivity of amiloride

For the FOREST assay, the biotinylated amiloride derivatives were immobilized on streptavidin magnetic beads and incubated with an RNA structure library identical to that used in our previous studies.^22,25^ This RNA library comprised 3000 distinct structured RNA motifs, including control sequences as well as RNAs derived from human precursor microRNAs (pre-miRNAs), viral RNAs, and repeat sequences. Following incubation, RNAs bound to the beads were collected and purified, and sequence analysis by barcode microarrays was performed. The fluorescence intensities obtained from the microarray scanning were standardized to calculate *Z*-score values, representing the relative binding affinities in the FOREST assay.

**Fig. 3:**
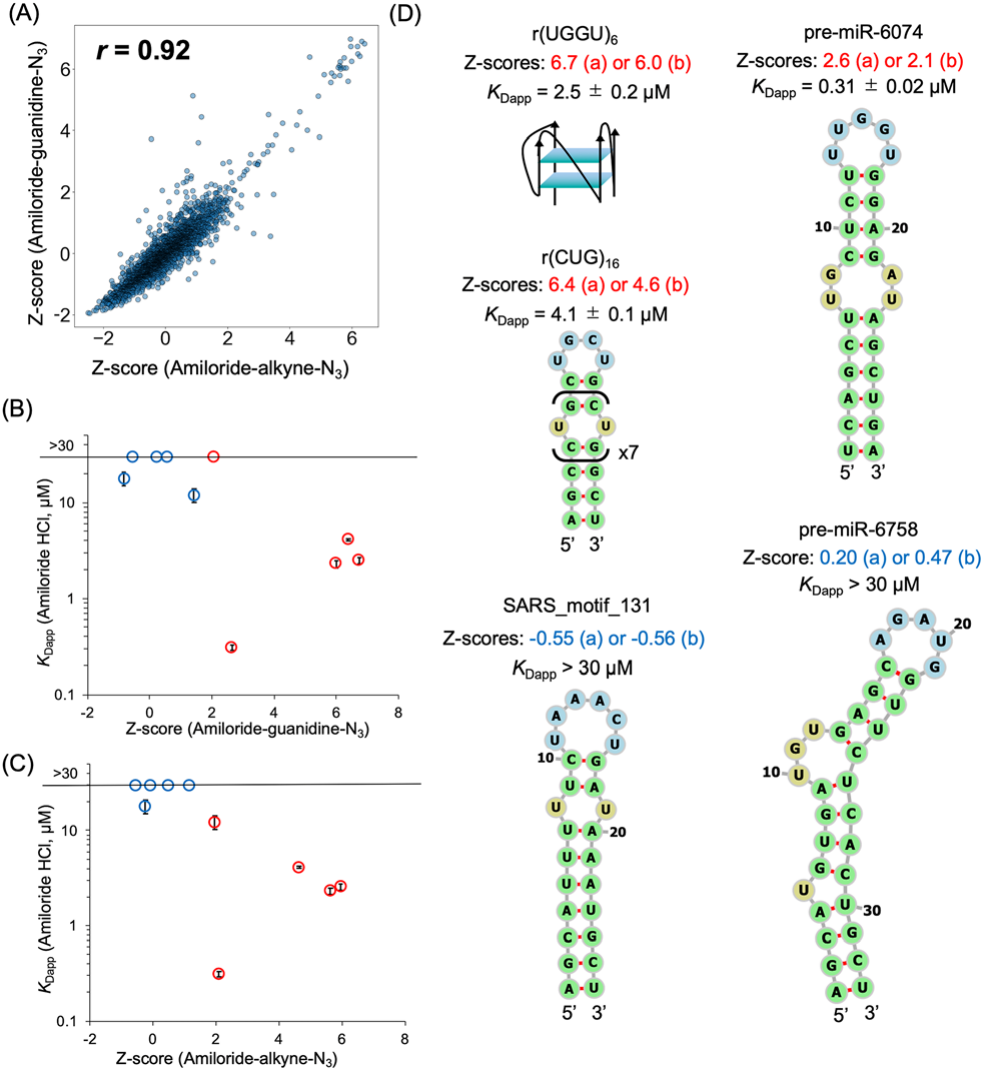
Large-scale analysis of the RNA-binding properties of amiloride using FOREST. (A) Correlation of *Z*-scores (relative binding scores by FOREST) between amiloride-guanidine-N_3_ and amiloride-alkyne-N_3_; *r* denotes Spearman's rank correlation coefficient. Relationship between *Z*-scores of (B) amiloride-guanidine-N_3_ or (C) amiloride-alkyne-N_3_ and apparent dissociation constants (*K*_Dapp_) measured by fluorescence titration experiments. Average *K*_Dapp_ values from two or three independent measurements are shown, with error bars representing standard errors. Red circles indicate high-ranking RNAs (*Z*-score > 1.96, *p* < 0.05), and blue circles indicate lower-ranking RNAs (*Z*-score ≤ 1.96). Pre-miR-627 and SARS-7171-Motif_3 were excluded from the graphs owing to negligible fluorescence changes. For SARS_motif_131, pre-miR-125a, and pre-miR-6758 (*K*_Dapp_ > 30 µM), *K*_Dapp_ values were set to 30 µM for graphing purposes. (D) Secondary structures of high- and intermediate-to-low-ranking RNAs with corresponding *K*_Dapp_ values. Structures were predicted using RNAfold, except for *r*(UGGU)_6_. Numbers below the name of the RNAs indicate *Z*-scores for (a) amiloride-guanidine-N_3_ and (b) amiloride-alkyne-N_3_. *K*_Dapp_ values from fluorescence titration are reported as mean ± standard error from three independent experiments, except SARS_motif_131 and pre-miR-6758 (*n* = 2).

For both amiloride derivatives, high-ranking sequences selected by *Z*-score thresholding (*Z*-score > 1.96, corresponding to a two-tailed *p* < 0.05) were predominantly enriched with RNAs containing multiple consecutive guanines (Supplementary Data 1 and 2 for amiloride-guanidine-N_3_ and amiloride-alkyne-N_3_, respectively), indicating a preference of the amiloride derivatives for binding to G-quadruplex (G4) RNAs. Importantly, in addition to G4 RNAs, the FOREST analysis revealed enrichment of RNAs containing U–U mismatches among the high-ranking sequences, including the toxic repeat RNA *r*(CUG)_16_, which is a potential target for small molecules.^26–28^ Furthermore, comparison of the *Z*-score values showed a strong correlation between the two amiloride derivatives (*r* = 0.92; Fig. 3A), indicating that despite differences in linker attachment positions, preservation of the hydrogen-bonding face and the guanidino group resulted in highly similar RNA-binding properties for both compounds.

Next, we attempted to validate the FOREST-derived rankings by fluorescence titration experiments using the synthesized amiloride derivatives and representative RNAs spanning the ranking spectrum, including G4 RNAs, pre-miRNAs, and SARS-CoV-2-derived RNAs. However, since a time-dependent decrease in the fluorescence of amiloride-guanidine-N_3_ during preliminary measurements was observed (data not shown), we instead evaluated whether the RNA-binding information obtained by FOREST using the amiloride derivatives reflected the intrinsic RNA-binding characteristics of linker-free amiloride. Accordingly, fluorescence titration experiments were performed using amiloride hydrochloride by monitoring changes in the fluorescence intensity of amiloride hydrochloride upon the addition of increasing amounts of RNAs, and the apparent dissociation constants (*K*_Dapp_) were determined (Fig. 3B–D and Fig. S2, S3, Table S1). These experiments revealed moderate binding affinities between amiloride and G4-containing RNAs, with *K*_Dapp_ values of 2.5 ± 0.2 µM for *r*(UGGU)_6_ and 2.3 ± 0.2 µM for G4_2A2_BCL2. Because amiloride can adopt a planar conformation through intramolecular hydrogen bonding, we speculate that amiloride interacts with G4 structures in a manner similar to that of previously reported cationic and planar small molecules.^29^

In addition to G4 RNAs, fluorescence titration experiments confirmed that amiloride binds to *r*(CUG)_16_ with moderate affinity (*K*_Dapp_ = 4.1 ± 0.1 µM), supporting the ability of amiloride to recognize U–U mismatch motifs. However, the most intriguing sequence identified was pre-miR-6074, which appeared among the high-ranking sequences in the FOREST analysis (rank 52 for amiloride-guanidine-N_3_ and 59 for amiloride-alkyne-N_3_). Although this sequence ranked lower than the G4 RNAs in the FOREST assay, subsequent fluorescence titration experiments, which are a solution-phase analysis, revealed an exceptionally high binding affinity (*K*_Dapp_ = 0.31 ± 0.02 µM), which is over 10-fold stronger than that for *r*(CUG)_16_. This result indicates that, although the bead-bound environment in the FOREST assay may have underestimated the affinity of pre-miR-6074 relative to G4 RNAs, possibly due to the effects of immobilization and linker attachment, FOREST nonetheless successfully captured the interaction between the amiloride derivatives and pre-miR-6074. In comparison, affinities between amiloride and lower-ranking RNAs (*Z*-score ≤ 1.96) were weak (*K*_Dapp_ > 30 µM), indicating that the RNA-binding information obtained from the FOREST assay using amiloride derivatives reflects the RNA-binding characteristics of amiloride. Moreover, several low-ranking RNAs contain U residues in the single-stranded regions, including a U–U mismatch in SARS_motif_131, yet only weak binding was observed.

### Effect of the linker on binding to pre-miR-6074

**Fig. 4:**
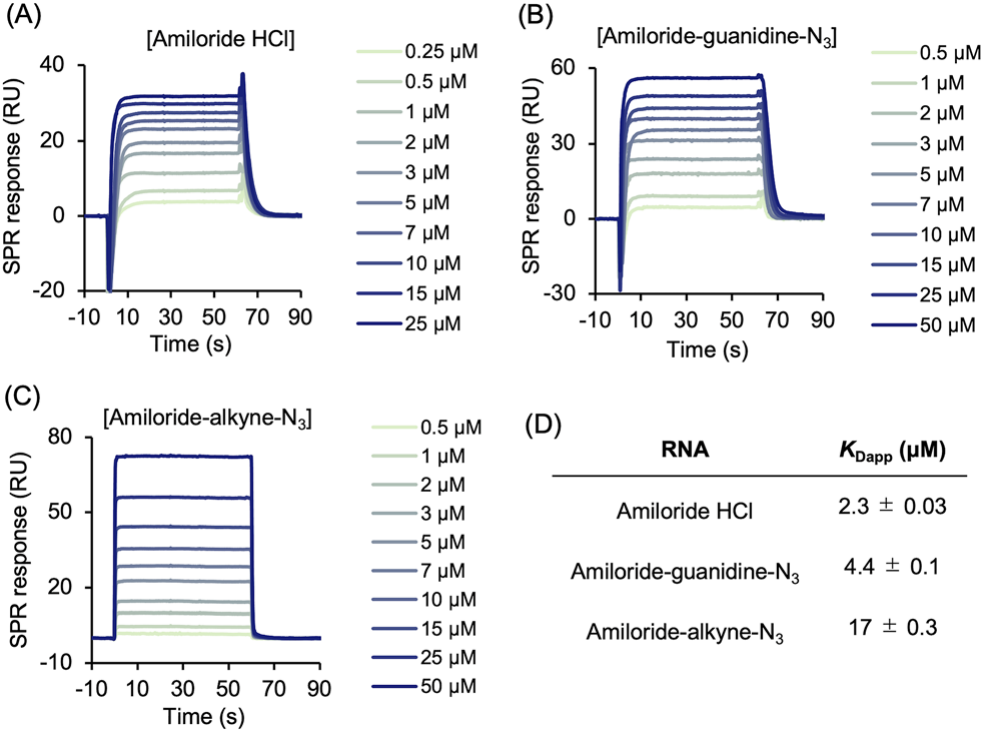
Surface plasmon resonance (SPR) analysis of amiloride and its derivatives. Representative SPR sensorgrams showing binding of (A) amiloride HCl, (B) amiloride-guanidine-N_3_, and (C) amiloride-alkyne-N_3_ to immobilized pre-miR-6074. (D) Summary of *K*_Dapp_ values obtained by SPR. Data are reported as mean ± standard error from three technical replicates.

Using pre-miR-6074, which exhibited unexpectedly strong affinity for amiloride in the fluorescence titration experiments, we next evaluated the effects of linker modifications on the interaction between the amiloride moiety and the RNA by SPR analysis. Upon the addition of amiloride compounds to pre-miR-6074 immobilized on a sensor chip, the SPR response increased in a concentration-dependent manner, which was well fitted to a steady-state affinity model to determine the apparent dissociation constants (*K*_Dapp_). The affinity decreased in the order of amiloride (*K*_Dapp_ = 2.3 ± 0.03 µM), amiloride-guanidine-N_3_ (*K*_Dapp_ = 4.4 ± 0.1 µM), and amiloride-alkyne-N_3_ (*K*_Dapp_ = 17 ± 0.3 µM) (Fig. 4 and Fig. S4). These results indicate that alkyne modification at the pyrazine ring negatively affects the interaction between the amiloride moiety and pre-miR-6074. Additionally, when methyl 3,5-diamino-6-chloropyrazine-2-carboxylate, an amiloride analogue lacking the guanidino group, was used as an analyte (Fig. S5), only a weak SPR response was observed (*K*_Dapp_ > 50 µM). This result indicates that the cationic guanidino group plays a critical role in binding, likely through electrostatic interactions with the negatively charged phosphate backbone of the RNA.^30,31^ It should also be noted that there are differences in the binding affinities obtained from the different assays. The specific experimental conditions and the obtained *K*_Dapp_ values are summarized in Table S3. The ∼10-fold discrepancy in the *K*_Dapp_ values for amiloride between the fluorescence titration and SPR assays likely arises from the fundamental differences in the assay environments. Specifically, while the fluorescence titration experiments were performed in bulk solution, SPR involves surface immobilization of the RNA, which may restrict the conformational freedom of the RNA and introduce steric constraints.

### Investigation of the binding site of amiloride on pre-miR-6074

**Fig. 5:**
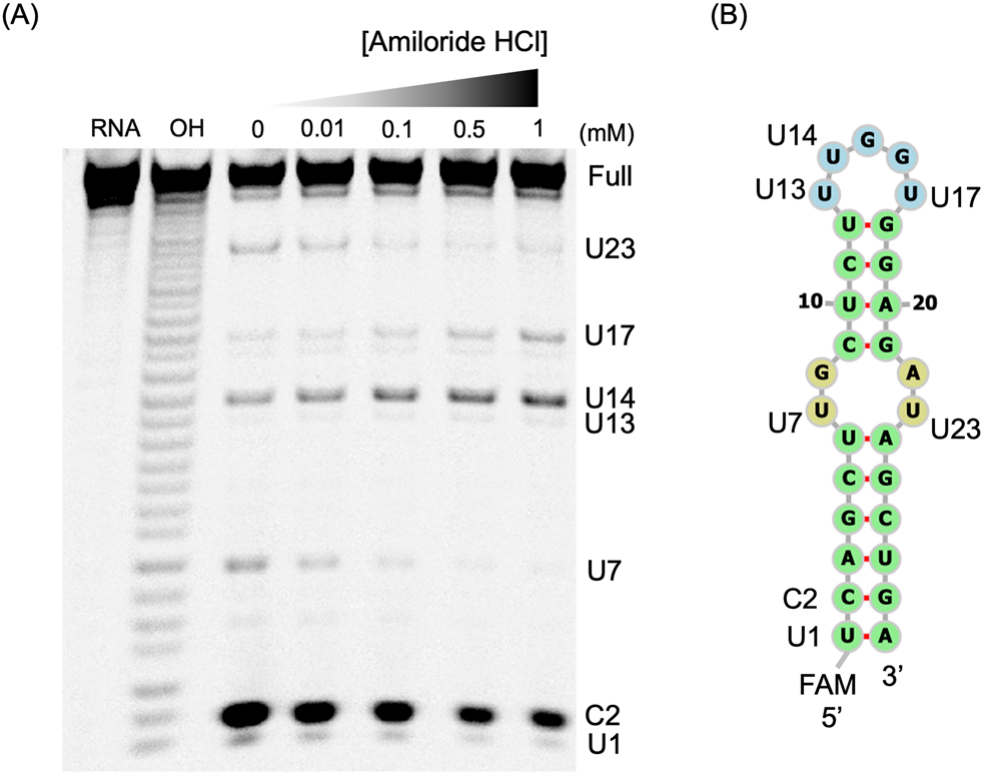
Investigation of the binding site by RNase A footprinting assay. (A) Gel image of the footprinting assay with amiloride HCl. Footprinting assay conditions: pre-miR-6074-FAM (1 µM), amiloride HCl (0–1 mM), RNase A (5 ng mL^−1^), buffer: 1% DMSO, 20 mM phosphate pH 7.0, 80 mM KCl, 20 mM NaCl, reaction time: 15 min at 37 °C. Lanes: pre-miR-6074 only (RNA), alkaline hydrolysis ladder (OH). The electrophoresis was performed on a 16% denaturing polyacrylamide gel containing 20% formamide. (B) Secondary structure of 5′-FAM-labeled pre-miR-6074 predicted by RNAfold.

Next, to identify the binding site of amiloride on pre-miR-6074, a footprinting assay using RNase A,^32^ which selectively cleaves RNAs at single-stranded pyrimidine residues, was performed. The results showed that cleavage at the internal loop was suppressed by amiloride in a concentration-dependent manner, whereas the cleavage at the hairpin loop was unaffected, indicating that the primary binding site of amiloride on pre-miR-6074 is the internal loop structure (Fig. 5). A similar trend was observed in an experiment using RNase T1, which cleaves RNAs at single-stranded guanine residues, further supporting the proposed binding position (Fig. S6). Additionally, to confirm the binding site of amiloride and to elucidate the structural specificity of pre-miR-6074 among other RNAs with U–U mismatches, fluorescence titration experiments using mutants of pre-miR-6074 were performed (Fig. S7). Upon addition of the pre-miR-6074_UU_mut, which lacks the U–U mismatch in the internal loop, to the amiloride solution, the fluorescence intensity of amiloride remained unchanged, in contrast to the quenching observed with the original sequence (*K*_Dapp_ = N.D.). This result indicates not only the binding site but also that the U–U mismatch in the internal loop is essential for the binding. Meanwhile, a mutant lacking the G–A mismatch in the internal loop (pre-miR-6074_GA_mut) exhibited reduced affinity for amiloride (*K*_Dapp_ = 3.2 ± 1 µM). This result suggests that the G–A mismatch may create an enlarged cavity that accommodates amiloride.

Furthermore, to assess whether amiloride influences the thermal stability of pre-miR-6074, melting temperature (*T*_m_) analysis was performed (Fig. S8). Amiloride neither stabilized nor destabilized the RNA structure (Table S3), consistent with our previous observation with G-clamp, which also did not affect the stability of its target RNA.^24^

### X-ray crystallographic analysis of the amiloride–RNA complex

**Fig. 6:**
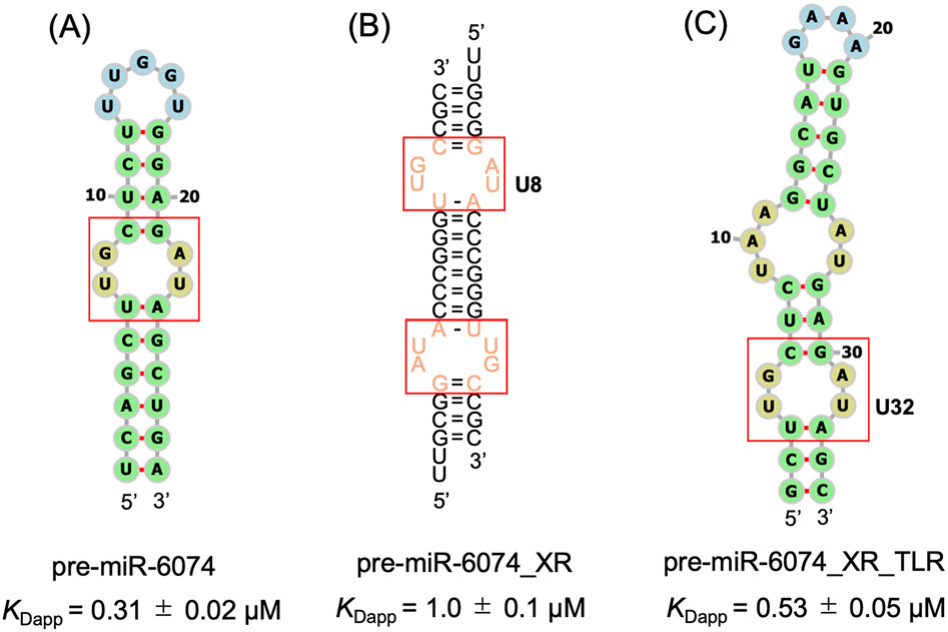
Design of RNA sequences for X-ray crystallographic analysis. Predicted secondary structures of (A) pre-miR-6074, (B) pre-miR-6074_XR, and (C) pre-miR-6074_XR_TLR, generated using RNAfold or RNAcofold. Red boxes indicate the extracted internal loop motif for the structural studies. The *K*_Dapp_ values determined by fluorescence titrations are reported as mean ± standard error from three independent measurements.

Based on the collected information, two RNAs were designed for X-ray crystallography using a motif extraction strategy to elucidate the binding mode of amiloride (Fig. 6). For both sequences, the region surrounding the internal loop of pre-miR-6074 was extracted and incorporated into model sequences. Specifically, pseudo-self-complementary pre-miR-6074_XR was designed based on our previous study, which utilized the ribosomal A-site model sequence to facilitate crystallization and structure determination.^24,33^ For pre-miR-6074_XR_TLR, the extracted internal loop motif was embedded into a tetraloop/tetraloop receptor model sequence, which is known to self-assemble and promote crystallization.^34^ The affinity of amiloride for the RNAs designed for X-ray crystallography was slightly reduced (*K*_Dapp_ = 1.0 ± 0.1 µM for pre-miR-6074_XR and *K*_Dapp_ = 0.53 ± 0.05 µM for pre-miR-6074_XR_TLR) compared to the original sequence, as determined by fluorescence titration experiments (Fig. S9).

The designed RNA sequences were crystallized in the presence of amiloride. A total of three crystals were obtained for both sequences, and the structures were solved at resolutions of 2.8 Å for pre-miR-6074_XR and 2.9 Å and 2.6 Å for pre-miR-6074_XR_TLR (forms 1 and 2, respectively) (Table S2). Unexpectedly, the structure of pre-miR-6074_XR was obtained in the absence of bound amiloride, presumably owing to the crystal packing structure of the RNA molecules or the reduced affinity of amiloride for this sequence. Therefore, this structure should be interpreted with caution, as the observed conformation may be influenced by crystal packing effects and the absence of the ligand. However, this structure revealed a cavity oriented toward the hydrogen-bonding edge of U8, suggesting the presence of a potential binding cavity that could accommodate ligand binding, rather than providing definitive evidence of the binding mode (Fig. S11).

**Fig. 7:**
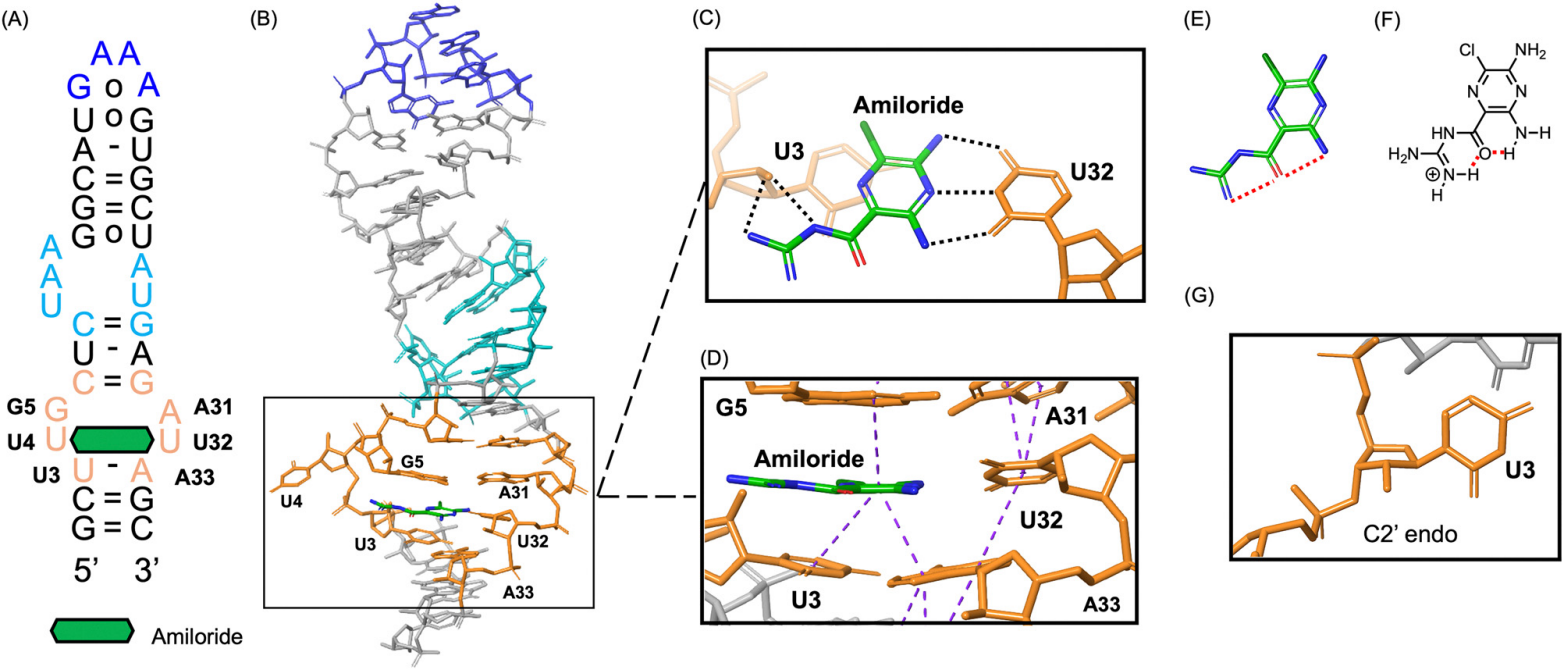
Crystal structure of amiloride in complex with pre-miR-6074_XR_TLR (form 1). (A) Secondary structure of pre-miR-6074_XR_TLR bound to amiloride, which is depicted as a green hexagon. Bases highlighted in orange correspond to the internal loop motif extracted from the original pre-miR-6074 sequence, dark blue denotes the tetraloop motif, and light blue indicates the tetraloop receptor motif. (B) Overall view of the pre-miR-6074_XR_TLR–amiloride complex. Close-up views of the binding site are shown from (C) the top and (D) the side, where black dashed lines represent intermolecular hydrogen bonds and purple dashed lines indicate stacking interactions. The conformation of amiloride observed in the crystal structure is shown in (E) stick representation and (F) chemical structure, with red dashed lines indicating intramolecular hydrogen bonds. (G) Conformation of U3 observed in the crystal structure.

On the other hand, for pre-miR-6074_XR_TLR, amiloride was bound to the extracted internal loop motif as expected (Fig. 7A and B). The 2mFo-DFc map clearly shows electron density corresponding to amiloride, supporting reliable placement of the ligand within the binding site (Fig. S12). In the crystal structure, amiloride was positioned in a geometry consistent with the formation of three hydrogen bonds with the O2, the imino hydrogen of N3, and the O4 of U32 (Fig. 7C). In addition to the pseudo base pair formation which is commonly observed in previously reported RNA–ligand complexes,^35,36^ the 2′-hydroxyl group of the neighboring U3 is likely to form a hydrogen bond with the guanidino group of amiloride, which further contributes to the strong binding. Moreover, stacking interactions between amiloride and the flanking U3, G5, and A33 residues were observed (Fig. 7D). This favorable stacking interaction is attributed to the highly planar conformation of amiloride, which is rigidified by intramolecular hydrogen bonds formed between the pyrazine amine and the carbonyl group, as well as between the carbonyl group and the guanidino group (Fig. 7E and F). Additionally, the B-factors of amiloride are comparable to those of the surrounding RNA residues, supporting stable ligand binding within the structure. Notably, across all determined crystal structures, the U residue neighboring the internal loop (U16 in pre-miR-6074_XR and U3 in pre-miR-6074_XR_TLR) consistently adopted a C2′-*endo* conformation, which is distinct from the canonical C3′-*endo* conformation typically observed in RNA (Fig. 7G).^37^ This specific conformation appears to pre-organize the binding pocket, thereby facilitating the binding of amiloride.

## Conclusions

In summary, this study provides a comprehensive evaluation of the RNA-binding properties of amiloride, a molecule characterized by its unique ability to form complementary hydrogen bonds with U. By leveraging the FOREST platform, we successfully identified naturally occurring RNA structural motifs that specifically interact with amiloride, including G4 RNAs and certain RNAs that contain U–U mismatches in their internal loops. Several pioneering studies have previously demonstrated the targeting of RNAs with internal loops and U-containing motifs using small molecules.^7,26–28,38^ Building upon these foundational concepts, the present study provides crucial atomic-resolution evidence detailing how a specific drug-like scaffold selectively engages a particular U–U mismatch. X-ray crystallographic analysis of the amiloride/pre-miR-6074_XR_TLR complex revealed that this high-affinity binding was achieved through a characteristic triple hydrogen-bonding pattern with U, complemented by stacking interactions and a unique C2′-*endo* ribose conformation at the binding site.

Importantly, these integrated biochemical and structural data reveal a clear mechanistic distinction in how amiloride engages different RNA targets. Its planar, cationic scaffold confers a general affinity for highly structured RNAs, such as G-quadruplexes with large stacking surfaces, primarily *via* electrostatic and stacking interactions. Building upon these basal interactions, target selectivity within the U–U mismatch pocket is strictly dictated by an additional, precise hydrogen-bonding network. Distinguishing this highly specific, hydrogen-bond-driven recognition from general electrostatic affinity provides a critical structural foundation for designing highly selective RNA-targeted therapeutics based on classic drug scaffolds.

Furthermore, the atomic-resolution structures provide an accurate framework for structure-based molecular design.^39^ In this work, structural analysis revealed that the guanidino group of amiloride is positioned in the shallow and relatively open minor groove of the RNA, providing a structural rationale for why modification at this site was well tolerated, as observed in the SPR analysis. Moreover, this positioning suggests that the guanidino group is an ideal site for the attachment of additional binding moieties or bulky fluorophores because its location in the minor groove permits chemical modifications without disrupting the essential hydrogen-bonding face required for U recognition. Building on our previous success with TO-G-clamp analogs, the insights gained in this study will enable the rational transformation of the amiloride scaffold into fluorogenic indicators. Such probes have the potential to serve as powerful analytical tools, including the visualization of specific RNA structures in cells^40^ and the high-throughput discovery of novel ligands targeting specific RNA motifs through FID assays.^41,42^ Based on these findings, we are currently further optimizing this scaffold and expanding its application to a broader range of functional RNA elements, with the goal of developing both RNA-targeting therapeutics and advanced analytical platforms.

## Author contributions

K. T., K. O., R. N. and J. K. designed the experiments. K. O. and F. N. supervised the research. K. T. synthesized the compounds. K. T., E. M., K. R. K. and H. S. performed the FOREST experiments. M. O. and J. K. performed X-ray crystallographic analysis. K. T. conducted biochemical and biophysical assays. K. T., K. O. and F. N. analyzed the results. K. T., K. O. and J. K. mainly wrote the manuscript. All authors discussed the results and provided feedback on the study and manuscript.

## Conflicts of interest

K. R. K. and H. S. own shares of xFOREST Therapeutics Co., Ltd. All other authors declare no competing financial interests.

## Supplementary Material

CB-007-D6CB00055J-s001

CB-007-D6CB00055J-s002

CB-007-D6CB00055J-s003

## Data Availability

The atomic coordinates and structure factors have been deposited in the Protein Data Bank (PDB) under accession codes 23JY (pre-miR-6074_XR), 23JZ (pre-miR-6074_XR_TLR Form 1), and 23KA (pre-miR-6074_XR_TLR Form 2). All other data supporting the findings of this study are available within the article and its supplementary information (SI). Supplementary information is available. See DOI: https://doi.org/10.1039/d6cb00055j. Ref. 43–50 are cited in the SI.
