# Supplementary material for: RNA-binding landscape of amiloride: large-scale profiling and structural basis of U–U mismatch recognition
Source: RSC Chem Biol. 2026 Apr 15;7(6):998–1005. doi: 10.1039/d6cb00055j (PMC13147285; doi:10.1039/d6cb00055j)
Supplement: CB-007-D6CB00055J-s001 [file CB-007-D6CB00055J-s001.pdf]

Supplementary Information (SI) for RSC Chemical Biology.
This journal is © The Royal Society of Chemistry 2026

Supplementary Information

RNA-binding Landscape of Amiloride: Large-scale Profiling and Structural Basis of U–U Mismatch Recognition

Kosuke Tsuzuki, [a,b] Kazumitsu Onizuka,*[a,b,c] Momo Okada,[d] Ryosuke Nagasawa,[a,b] Emi Miyashita,[e] Kaoru R. Komatsu,[e] Hirohide Saito,[e,f] Jiro Kondo,*[d] and Fumi Nagatsugi*[a.b]

[a] Institute of Multidisciplinary Research for Advanced Materials, Tohoku University, 2-1-1 Katahira, Aoba-ku, Sendai, Miyagi 980-8577, Japan.

[b] Department of Chemistry, Graduate School of Science, Tohoku University, Aoba-ku, Sendai 980-8578, Japan.

[c] Division for the Establishment of Frontier Sciences of Organization for Advanced Studies, Tohoku University, Aoba-ku, Sendai, Miyagi 980-8577, Japan.

[d] Department of Materials and Life Sciences, Faculty of Science and Technology, Sophia University, Tokyo 102-8554, Japan.

[e] Center for iPS Cell Research and Application (CiRA), Kyoto University, 53 Kawahara-cho, Shogoin, Sakyoku, Kyoto, 606-8507, Japan.

[f] Institute for Quantitative Biosciences, The University of Tokyo, Tokyo 113-0032, Japan

Table of Contents

Experimental procedure p2-p7
Supplementary tables (Table S1-S3) p8-p9
Supplementary figures (Figure S1-S12) p10-p15
Synthesis (Schemes S1 and 2) and NMR spectra p16-p24
References p25

Experimental procedure

Materials and Methods

General chemicals and solvents were purchased from FUJIFILM Wako Pure Chemical, Tokyo Chemical Industry, Kanto Chemical, Nacalai-tesque and Sigma-Aldrich. RNAs were purchased from JBioS (Japan). ^{1}H NMR spectra (400, 500, and 600 MHz) were recorded using Bruker AVANCE III 400, 500 and 600 spectrometers. ^{13}C NMR spectra (126 and 151 MHz) were recorded using Bruker AVANCE III 500 and 600 spectrometers. High-resolution electrospray ionization mass (ESI-HRMS) analysis was performed using a Bruker MicrOTOF-Q II. HPLC purifications and analyses were performed with a JASCO HPLC System (CO-631A) using a reverse-phase C_{18} column (COSMOSIL 5C_{18}-AR-II, Nacalai Tesque, 10 × 250 mm for ligand purification and 4.6 × 250 mm for analysis).

Synthesis of Amiloride-guanidine-N_3 and Amiloride-alkyne-N_3 for FOREST analysis

Amiloride-guanidine-N_3 and Amiloride-alkyne-N_3 were synthesized according to Schemes 1 and 2, respectively.

Biotinylation of Amiloride-guanidine-N_3 by SPAAC reaction

To a solution of DBCO-SS-biotin in DMSO (15 mM, 1.2 µL), a solution of Amiloride-guanidine-N_3 in DMSO (16.6 mM, 1.2 µL) and DMSO (1.6 µL) were added, and the mixture was incubated at 37 °C. After 1 hour, the mixture was analyzed by reversed-phase HPLC. HPLC conditions; A: 0.1% TFA in distilled water, B: 0.1% TFA in MeCN; B: 5% → 60% (〜20 min) → 100% (〜25 min) → 100%(〜30 min). Flow rate = 1 mL/min; Temp. = 35 °C; UV = 254 nm, C-18 column (Nacalai Tesque: COSMOSIL 5C_{18}-AR-II, 4.6 × 250 mm). The mixture was used without any purification. Amiloride-guanidine-biotin: ESI-HRMS (*m*/*z*): $[M+2H]^{2+}$ calcd for $C_{56}H_{81}ClN_{16}O_{12}S_3^{2+}$, 650.2530; found 650.2543.

Biotinylation of Amiloride-alkyne-N_3 by SPAAC reaction

To a solution of DBCO-SS-biotin in DMSO (15 mM, 1.2 µL), a solution of Amiloride-alkyne-N_3 in DMSO (1.05 mM, 19 µL) and DMSO (0.8 µL) were added, and the mixture was incubated at 37 °C. After 1 hour, the mixture was analyzed by reversed-phase HPLC. HPLC conditions; A: 0.1% TFA in distilled water, B: 0.1% TFA in MeCN; B: 5% → 60% (〜20 min) → 100% (〜25 min) → 100%(〜30 min). Flow rate = 1 mL/min; Temp. = 35 °C; UV = 254 nm, C-18 column (Nacalai Tesque: COSMOSIL 5C_{18}-AR-II, 4.6 × 250 mm). The mixture was used without any purification. Amiloride-alkyne-biotin: ESI-HRMS (*m*/*z*): $[M+2H]^{2+}$ calcd for $C_{63}H_{91}N_{17}O_{14}S_3^{2+}$, 702.8042; found 702.8058.

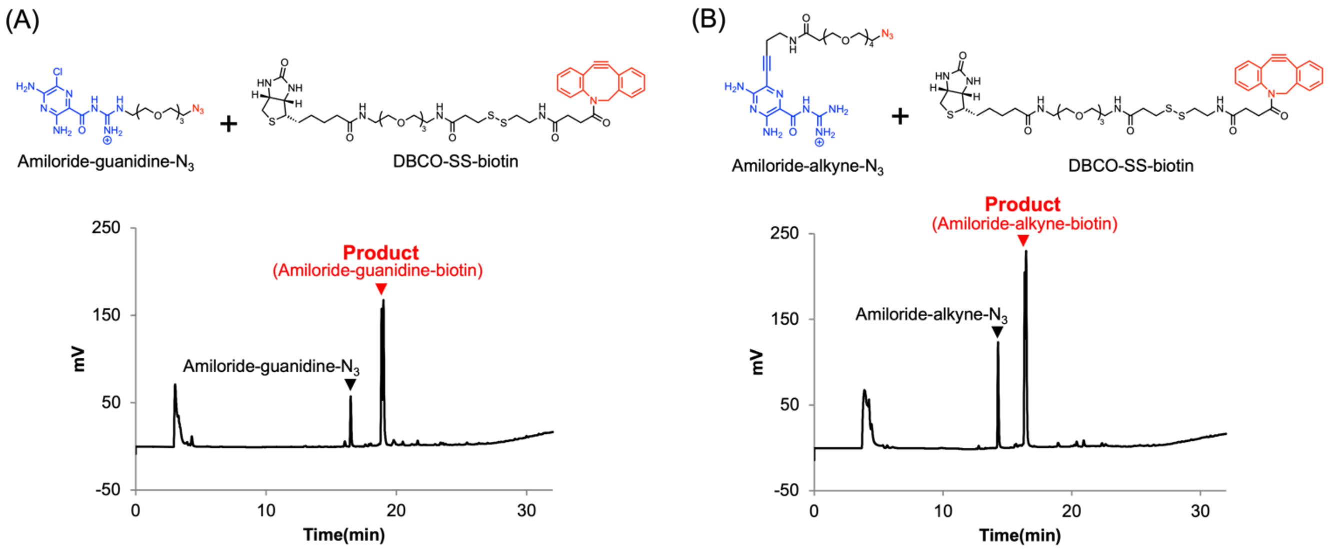

HPLC profiles of biotin conjugation to (A) Amiloride-guanidine-N_3, and (B) Amiloride-alkyne-N_3 by strain-promoted azide–alkyne cycloaddition (SPAAC).

FOREST assay

In this study, we used the same RNA structure library that was used in our previous study.(1) The preparation of the RNA structure library including *in silico* RNA motif extraction, design of a template pool of RNA structure library and DNA barcode microarray, and 3'-Terminal labeling with Cy3, has been reported in our previous studies.(1,2)

RNA pull-down

The experiment was conducted with slight modifications from the previously reported procedure.(1) The RNA structure library (1 ng/μL, 500 μL) in 1× Binding buffer (20 mM phosphate pH 7.0, 20 mM NaCl, and 80 mM KCl) was heated at 95 °C for 5 min, then cooled on ice to fold the RNAs. In a separate tube, Amiloride-guanidine-biotin or Amiloride-alkyne-biotin in DMSO (0.5 mM, 1 μL) and 50 μL of Streptavidin Mag Sepharose (Cytiva) were mixed in 900 μL of 1× Binding buffer to prepare the small molecule-conjugated streptavidin magnetic beads. In addition, Mock beads for background subtraction were prepared by mixing DMSO (1 μL) and 50 μL of Streptavidin Mag Sepharose in 1× Binding buffer. The beads mixture was vortexed at room temperature for 60 min. The tube was then placed on a magnetic rack and the supernatant was discarded, then the folded RNA structure library was added. The mixture was vortexed at room temperature for further 60 min. After the mixing, the supernatant containing unbound RNA was removed on a magnetic rack, and the mixture was washed three times with 200 μL of 1× Binding buffer. 200 μL of 1× Elution buffer (1% SDS (w/v), 10 mM Tris-HCl pH 8.0, 2 mM EDTA) was then added to the magnetic beads, and the mixture was heated at 95 °C for 3 min. The tube was placed on a magnetic rack and the supernatant containing bound RNA was collected. The collected RNA was purified with phenol-chloroform extraction and ethanol precipitation before the microarray scanning.

Hybridization and microarray scanning

Eighteen microliters of the bound RNA structures were mixed with 4.5 μL of 10× Blocking Agent (Agilent Technologies) and 22.5 μL of Hi-RPM Hybridization Buffer (Agilent Technologies). The samples were incubated for 5 min in a heat block set at 100 °C, then rapidly cooled and incubated for 5 min in ice water. The samples were applied to an 8 × 60K Agilent microarray gasket slide (Agilent Technologies). The prepared gasket slide and CGH custom array 8 × 60K (Agilent Technologies) were assembled with SureHyb. Hybridization was performed for 20 h

at a temperature of 55.5 °C at 20 rpm. The microarray slide was washed for 5 min with Gene Expression Wash Buffer 1 (Agilent Technologies) in a glass container at room temperature following hybridization. The microarray slide was moved to a glass container containing Gene Expression Wash Buffer 2 (Agilent Technologies), which was immersed in a thermostatic bath at 37 °C. The washing step was performed for 5 min. Fluorescence scanning was performed on the microarray, and fluorescence image data were acquired using SureScan (Agilent Technologies). The acquired images were converted to numeric fluorescence intensities for each spot by Feature Extraction (Agilent Technologies) and GeneSpringGX (Agilent Technologies).

Calculation of binding intensity

The binding intensities of each RNA structure were calculated by subtracting the fluorescence intensities of the no-ligand control samples. To alleviate the effect of undesired interactions with the RNA barcode, we calculated the mean fluorescence intensities of each RNA structure from the intensities of three RNA probes that had the same RNA structure but different RNA barcodes. To that end, maximum and the minimum values among the five barcodes were filtered.

Calculation of Z-scores

The binding strength is normalized as a *Z*-score using Eq. (1): μ is the mean value of the library population, σ is the standard deviation, and x is the binding intensity of each probe in the library.

$$\text{Z-score} = (x-\mu)/\ \sigma \quad (1)$$

High-ranking RNA motifs were selected using Z-score thresholding. A cutoff of Z-score > 1.96, corresponding to a two-tailed $p < 0.05$ level in a standard normal distribution, was applied.

RNA secondary structure prediction and visualization

The secondary structures of the RNAs were predicted by RNAfold and RNAcofold web servers.(3) The temperature was set to 25 °C for the predictions. The minimum free energy structures were used in this study and the Forna website was used to generate illustrations of the predicted secondary structures of the RNAs.(4)

Fluorescence titration

Fluorescence titration experiments were performed with a JASCO-6500 spectrofluorometer (JASCO, Tokyo, Japan). A solution (50 μL) of amiloride hydrochloride (100 nM) in 1× Fluorescence titration buffer (1% DMSO (v/v), 20 mM phosphate pH 7.0, 20 mM NaCl and 80 mM KCl) was prepared in a micro quartz cell with a 1 cm path length. Serial aliquots of a concentrated solution of folded RNA in 1× Binding buffer (20 mM phosphate pH 7.0, 20 mM NaCl and 80 mM KCl) were added to the solution in the cell and allowed to equilibrate for 2 or 5 min. The excitation wavelength was set at 381 nm and the emissions were recorded at 20 °C.

The data from the fluorescence titration experiments (fluorescence intensities at 420 nm) were analyzed using the independent-site model by non-linear fitting Equation (2), in which F is the measured fluorescence intensity, F_0 is the initial fluorescence intensity in the absence of RNA, Q (= F_{final}/F_0) is the fluorescence enhancement or diminution, A = K_{Dapp}/C_{ligand} and X = nC_{RNA}/C_{ligand} (n is the putative number of binding sites on RNA and n = 2 was used for pre-

miR-6074_XR and n = 1 was used for the other RNAs).(5) Two or three independent measurements were conducted.

$$\Delta F = F - F_0 = F_0(Q-1)/2\{A+1+X-[(X+1+A)^2-4X]^{1/2}\} \quad (2)$$

SPR analysis

SPR measurements were performed with Biacore T200 (Cytiva) and the data were analyzed by Biacore T200 evaluation software (Cytiva).

Immobilization: 5′-biotinylated RNA (pre-miR-6074-biotin) was diluted to 1 μM in 1× Binding buffer (20 mM phosphate pH 7.0, 20 mM NaCl, and 80 mM KCl), and the solution was heated at 95 °C for 5 min and rapidly cooled on ice. The folded RNAs were injected over a streptavidin-coated sensor chip (Series S Sensor chip SA, Cytiva) at 30 μL/min to reach an immobilization level of 1351 or 1374 RU.

Binding analysis: Analytes (amiloride hydrochloride, Amiloride-guanidine-N_3, Amiloride-alkyne-N_3, and methyl 3,5-diamino-6-chloropyrazine-2-carboxylate) in 1× SPR buffer (1% DMSO (v/v), 20 mM phosphate pH 7.0, 20 mM NaCl, and 80 mM KCl) were injected at increasing concentrations (0.25-25 μM for amiloride hydrochloride, 0.5-50 μM for Amiloride-guanidine-N_3, Amiloride-alkyne-N_3, and methyl 3,5-diamino-6-chloropyrazine-2-carboxylate), to the RNA-immobilized sensor chip surface with a regeneration step between each concentration. Each of the analytes in 1× SPR buffer was injected with a flow rate of 30 μL/min, contact time of 60 s, and dissociation time of 120 s using the SPR buffer at 25 °C. A regeneration step was conducted with a flow rate of 30 μL/min and contact time of 60 s using the 1× SPR buffer. All sensorgrams were corrected by subtracting the blank flow cell and buffer injection responses. The K_{Dapp} values were calculated using the steady state affinity model; [$y = \{(R_{max} \cdot x) / (K_D + x)\} + C$], where y is the SPR response, R_{max} is the maximum SPR response, K_{Dapp} is the apparent dissociation constant, x is the concentration of the added RNA binder, C is the offset value. Two or three technical replicates were conducted.

RNase footprinting assays

Ten micro-molar of 5’-FAM labeled pre-miR-6074 was folded in 1× Binding buffer (20 mM phosphate pH 7.0, 20 mM NaCl, and 80 mM KCl) by heating at 95 °C for 5 min and rapidly cooling on ice. To a solution of 0.5 μL of the folded RNA (10 μM), 1.25 μL of H_2O, 2.25 μL of 2× Binding buffer (40 mM phosphate pH 7.0, 40 mM NaCl, and 160 mM KCl), and 0.5 μL of amiloride hydrochloride solution (0-10 mM in 10% DMSO (v/v), H_2O) were added and incubated at 37 °C for 60 min. RNase T1 (2700 ng/mL, 0.5 μL, Worthington) or RNase A (50 ng/mL, 0.5 μL, Thermo Fisher Scientific) was added to the mixture, and samples were incubated at 37°C for an additional 15 min. The reactions were stopped by adding 5 μL of Gel Loading Buffer (80% (v/v) formamide, 10 mM EDTA). To generate the hydrolysis ladder (OH), 5’-FAM labeled pre-miR-6074 was hydrolyzed under an alkaline condition (45 mM sodium carbonate solution pH 10, 0.9 mM EDTA) for 5 min at 95 °C and rapidly cooling on ice. Cleavage products were resolved on a denaturing gel (16% (w/v) polyacrylamide, 20% (v/v) formamide, 7.5 M urea), which was visualized by ChemiDoc Touch MP gel imager (Bio-Rad).

Crystallization, X-ray data collection, structure determination, and structure refinement

Two RNA constructs were designed for X-ray crystallographic analysis to investigate the binding mode of amiloride. The internal loop motif of pre-miR-6074 was extracted and embedded into model RNA sequences using a motif

extraction strategy. As stated in the main text, pre-miR-6074_XR was designed as a pseudo-self-complementary duplex based on a ribosomal A-site model sequence. The other construct, pre-miR-6074_XR_TLR, was engineered by inserting the same motif into a tetraloop/tetraloop receptor (TLR) framework known to promote RNA self-assembly and crystallization.

Crystallization trials were performed at 293 K using vapor diffusion methods in the presence of amiloride. For pre-miR-6074_XR, crystallization was carried out using the sitting-drop vapor diffusion method. A 0.2 μL aliquot of amiloride-RNA solution (1 mM RNA, 2 mM amiloride) was mixed with 0.2 μL of crystallization solution containing 50 mM MOPS (pH 7.0), 10 mM hexammine cobalt chloride, 500 mM potassium chloride, and 10% (v/v) 2-methyl-2,4-pentanediol (MPD). The droplet was equilibrated against 30 μL of reservoir solution containing 40% (v/v) MPD.

For pre-miR-6074_XR_TLR, two distinct crystal forms were obtained under different conditions. Form 1 was obtained by hanging-drop vapor diffusion. A 1 μL aliquot of amiloride-RNA solution (0.25 mM RNA, 0.5 mM amiloride) was mixed with 1 μL of crystallization solution containing 50 mM sodium cacodylate trihydrate (pH 6.0), 200 mM potassium chloride, 10 mM calcium chloride dihydrate, and 10% (w/v) polyethylene glycol 4000. The drop was equilibrated against 250 μL of the crystallization solution in the reservoir. Form 2 was obtained by sitting-drop vapor diffusion. A 0.2 μL aliquot of amiloride-RNA solution (0.25 mM RNA, 0.5 mM amiloride) was mixed with 0.2 μL of crystallization solution containing 100 mM sodium cacodylate trihydrate (pH 6.5), 200 mM sodium citrate tribasic dihydrate, and 30% (v/v) 2-propanol. The drop was equilibrated against 30 μL of the crystallization solution in the reservoir.

Crystals were cryoprotected by transferring them into 40% (v/v) MPD, depending on the condition, and flash-frozen in liquid nitrogen. Diffraction data were collected at 100 K using synchrotron radiation. For pre-miR-6074_XR and pre-miR-6074_XR_TLR (Form 2), data were collected at beamline BL-17A of the Photon Factory (Tsukuba, Japan), while data for pre-miR-6074_XR_TLR (Form 1) were collected at beamline BL-5A. Diffraction images were processed using the XDS program. Initial phases were obtained by molecular replacement using Phaser from the Phenix suite. For pre-miR-6074_XR_TLR (Forms 1 and 2), the search model was derived from the tetraloop-tetraloop receptor complex (PDB ID: 4FNJ). For pre-miR-6074_XR, molecular replacement was performed using an ensemble of RNA models generated with 4MRNA (Massive Multi-type Model Molecular Replacement for Nucleic Acids), an original tool developed by S. Ando and J. Kondo (available at https://github.com/S-Ando-Biophysics/4MRNA). Model building and refinement were performed with Coot and phenix.refine, including restrained refinement and B-factor optimization. The maximum resolutions were 2.8 Å for pre-miR-6074_XR, 2.9 Å for pre-miR-6074_XR_TLR (Form 1), and 2.6 Å for Form 2. Data collection and refinement statistics are summarized in Table S2. Structural figures were prepared using PyMOL and MacroModel. The atomic coordinates and structure factors have been deposited in the Protein Data Bank under accession codes 23JY, 23JZ, and 23KA (pre-miR-6074_XR, pre-miR-6074_XR_TLR (Form 1), and pre-miR-6074_XR_TLR (Form 2), respectively).

The overall structures of the RNA molecules observed in the asymmetric units are shown in Figure S10. The crystal of pre-miR-6074_XR contains a single RNA duplex in the asymmetric unit. The pre-miR-6074_XR_TLR (Form 1) crystal also contains one RNA molecule per asymmetric unit, whereas the Form 2 crystal contains three RNA molecules. Despite these differences in the number of molecules per asymmetric unit, the four RNA structures obtained from Form 1 and Form 2 were highly similar, suggesting a conserved conformation of the TLR-embedded RNA in the presence of amiloride.

Melting temperature T_m analysis

T_m curves of folded pre-miR-6074 (1 μM) with amiloride hydrochloride (0, 1, and 5 μM) in 1× T_m buffer (1% DMSO, 20 mM phosphate pH 7.0, 20 mM NaCl, and 80 mM KCl) were recorded on a JASCO V-730 spectrophotometer (JASCO, Tokyo, Japan) equipped with a PAC-743 temperature controller and a cell with a 1 cm path length. The absorbance values at 260 nm were monitored while heating the samples from 20 °C to 95 °C with a heating rate of 1 °C /min. The absorbance values were normalized according to Equation (3), in which $Abs_{measured}$ is the measured absorbance value, Abs_{min} is the minimum absorbance value among the recorded data, and Abs_{max} is the maximum absorbance value among the recorded data.

$$\text{Normalized absorbance} = (Abs_{measured} - Abs_{min}) / (Abs_{max} - Abs_{min}) \quad (3)$$

T_m curves were plotted based on the normalized absorbance values, and the T_m values were determined based on the normalized absorbance values, using the second derivative method. Three independent measurements were conducted.

Table S1. Summary of RNA sequences used in this study and the apparent dissociation constants (K_{Dapp}) between the RNAs and amiloride as determined by fluorescence titrations.

Name	Sequence (5'- 3')	Rank (Amiloride-guanidine-N_3)	Rank (Amiloride-alkyne-N_3)	K_{Dapp} (μM)
r(UGGU)$_6$	AGCUGGUUGGUUGGUUGGUUGGUUGGUGCU	5	7	2.5 ± 0.2[a)]
r(CUG)$_{16}$	AGCCUGCUGCUGCUGCUGCUGCUGCUGCUGCUGCUGCUGCUGCUGCUGGCU	8	25	4.1 ± 0.1[a)]
G4_2A2_BCL2	AGCAAGGGGGCCGUGGGGUGGGAGCUGGGGAAGCU	17	13	2.3 ± 0.2[a)]
pre-miR-6074	UCAGCUUGCUCUUUGGUGGAGAUAGCUGA	52	59	0.31 ± 0.02[a)]
pre-miR-6074_UU_mut	UCAGCUGCUCUUUGGUGGAGAAGCUGA	-	-	N.D.[b)]
pre-miR-6074_GA_mut	UCAGCUUGCUCUUUGGUGGAGCUAGCUGA	-	-	3.2 ± 1
pre-miR-6074_XR	UUGCGGAUACCCGGGUUGCCGC	-	-	1.0 ± 0.1[a)]
pre-miR-6074_XR_TLR	GCUUGCUCUAAGGCAUGAAAGUGCUAUGAGAUAGC	-	-	0.53 ± 0.05[a)]
pre-miR-5008	AGCCAGUGGUGCCAUCUCCCCUGGCU	70	1520	> 30[c)]
pre-miR-1256	AGCAGCUGUGAAAGUCCUAGCUGCU	162	68	12 ± 2[a)]
SARS_7171_motif3	AGCCUAGGUUUUUCUAUGUACUUGGGCU	490	341	N.D.[b)]
pre-miR-627	AGCAAGAGGAGGUGGUUGUUUUCCUCCUCUUGCU	815	680	N.D.[b)]
pre-miR-125a	AGCCUGUGAGGACAUCCAGGGUCACAGGCU	639	251	> 30[c)]
SARS_motif_131	AGCAUUUUUCUAAACUGAUAAAUGCU	2172	2179	> 30[c)]
pre-miR-6758	AGCAUGUGAUGUGAGCAGAUGGUUCUCACUGCU	1067	795	> 30[c)]
pre-miR-548ab	AGCUGCUAUUACUUGUAUUUAUUUGUAAUGCAGCU	2518	1731	18 ± 1[a)]

a) Data are reported as mean ± standard error from three independent measurements. b) Data are reported as N.D. due to negligible change upon the addition of RNA (n =2). c) Data are mean of two measurements.

Table S2. Crystal data and statistics of data collection and structure refinement

Crystal code	pre-miR-6074_XR	pre-miR-6074_XR_TLR/amiloride complex (Form 1)	pre-miR-6074_XR_TLR/amiloride complex (Form 2)
Crystal data			
Space group	$P6_122$	$P3_121$	$P3_1$
Unit cell (Å)	a = b = 66.6, c = 53.3	a = b = 65.1, c = 69.5	a = b = 61.6, c = 106.2
No. of Molecules in AU [a]	1	1	3
Data collection			
Beamline	BL-17A of PF	BL-5A of PF	BL-17A of PF
Wavelength (Å)	0.98	0.98	0.98
Resolution (Å)	28.9-2.8	29.6-2.9	37.6-2.6
of the outer shell (Å)	2.9-2.8	2.98-2.9	2.66-2.59
Unique reflections	1782	3993	13907
Completeness (%)	92.2	99.8	99.9
in the outer shell (%)	82.6	101.4	98.6
R_{merge} [b] (%)	8.0	12.0	6.5
in the outer shell (%)	30.8	38.2	34.3
Redundancy	4.6	4.7	10.5
in the outer shell	3.8	5.0	10.1
I/σ(I)	11.7	11.6	23.5
in the outer shell	4.51	4.1	4.8
Structure refinement			
Resolution range (Å)	28.9-2.8	29.6-2.9	37.6-2.6
Used reflections	3214	3985	13903
R-factor [c] (%)	19.9	19.5	18.2
R_{free} [d] (%)	25.8	23.7	21.8
R.m.s.d. bond length (Å)	0.009	0.006	0.008
R.m.s.d. bond angles (°)	1.6	1.1	1.6

[a] Number of nucleic acid strands in the asymmetric unit.
[b] $R_{merge} = 100 \times \Sigma_{hklj}|I_{hklj} - \langle I_{hklj}\rangle| / \Sigma_{hklj}\langle I_{hklj}\rangle$.
[c] R-factor = $100 \times \Sigma||F_o| - |F_c|| / \Sigma|F_o|$, where $|F_o|$ and $|F_c|$ are optimally scaled observed and calculated structure factor amplitudes, respectively.
[d] Calculated using a random set containing 10% of observations.

Table S3. Summary of the biochemical assay conditions. The detailed experimental conditions and data analysis methods are reported in the Experimental procedure section.

	**Fluorescence titration**	**SPR analysis**	**T_m analysis**
**Assay type**	Affinity	Affinity	Thermal stability
**RNA & ligand**	pre-miR-6074 & Amiloride HCl	pre-miR-6074-biotin & Amiloride HCl	pre-miR-6074 & Amiloride HCl
**Term**	K_{Dapp} (Apparent dissociation constant)	K_{Dapp} (Apparent dissociation constant)	T_m (Melting temperature)
**Measured values**	0.31 ± 0.02 μM	2.3 ± 0.03 μM	62.3 ± 0.2 ℃ (Amiloride HCl 0 eq)<br>62.2 ± 0.2 ℃ (Amiloride HCl 1 eq)<br>62.3 ± 0.2 ℃ (Amiloride HCl 5 eq)
**Buffer**	1% DMSO (v/v), 20 mM Phosphate pH 7.0, 80 mM KCl, 20 mM NaCl		
**Temperature**	20 ℃	25 ℃	20 ℃ ↗ 95 ℃ (Heating rate: 1 ℃ /min)
**Immobilization status (RNA)**	Free	Immobilized	Free
**Immobilization status (ligand)**	Free	Free	Free

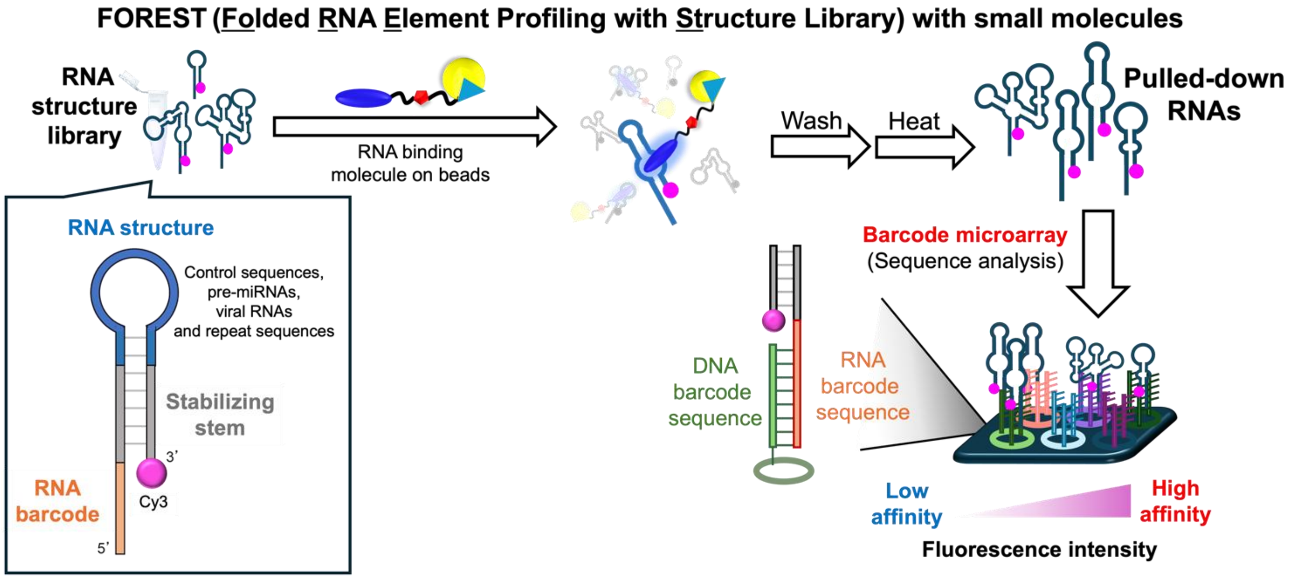

Figure S1. Illustration of using FOREST to analyze small-molecule RNA interactions.

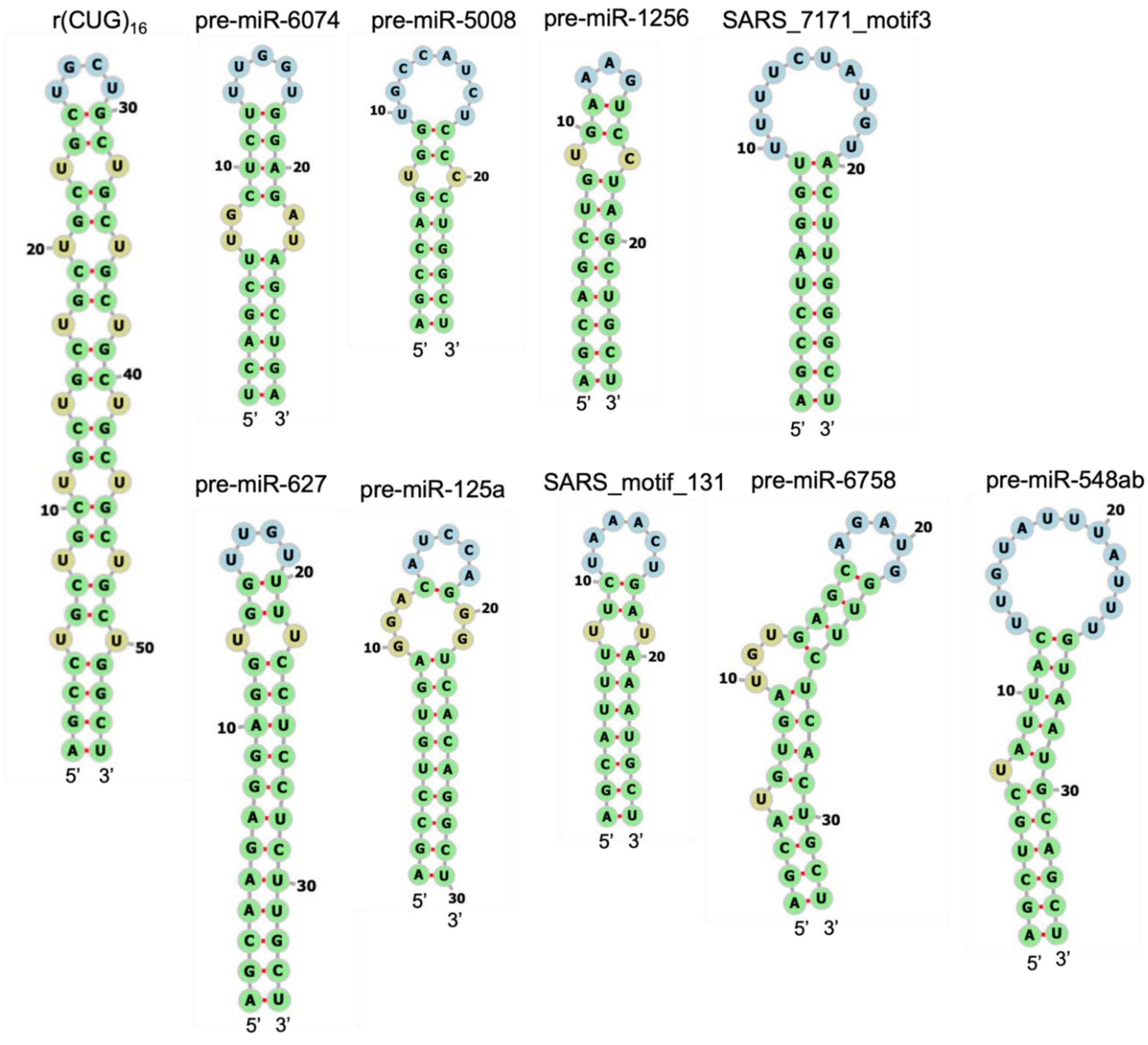

Figure S2. Secondary structures of the RNAs used for the validation of FOREST. The structures were predicted by RNAfold.

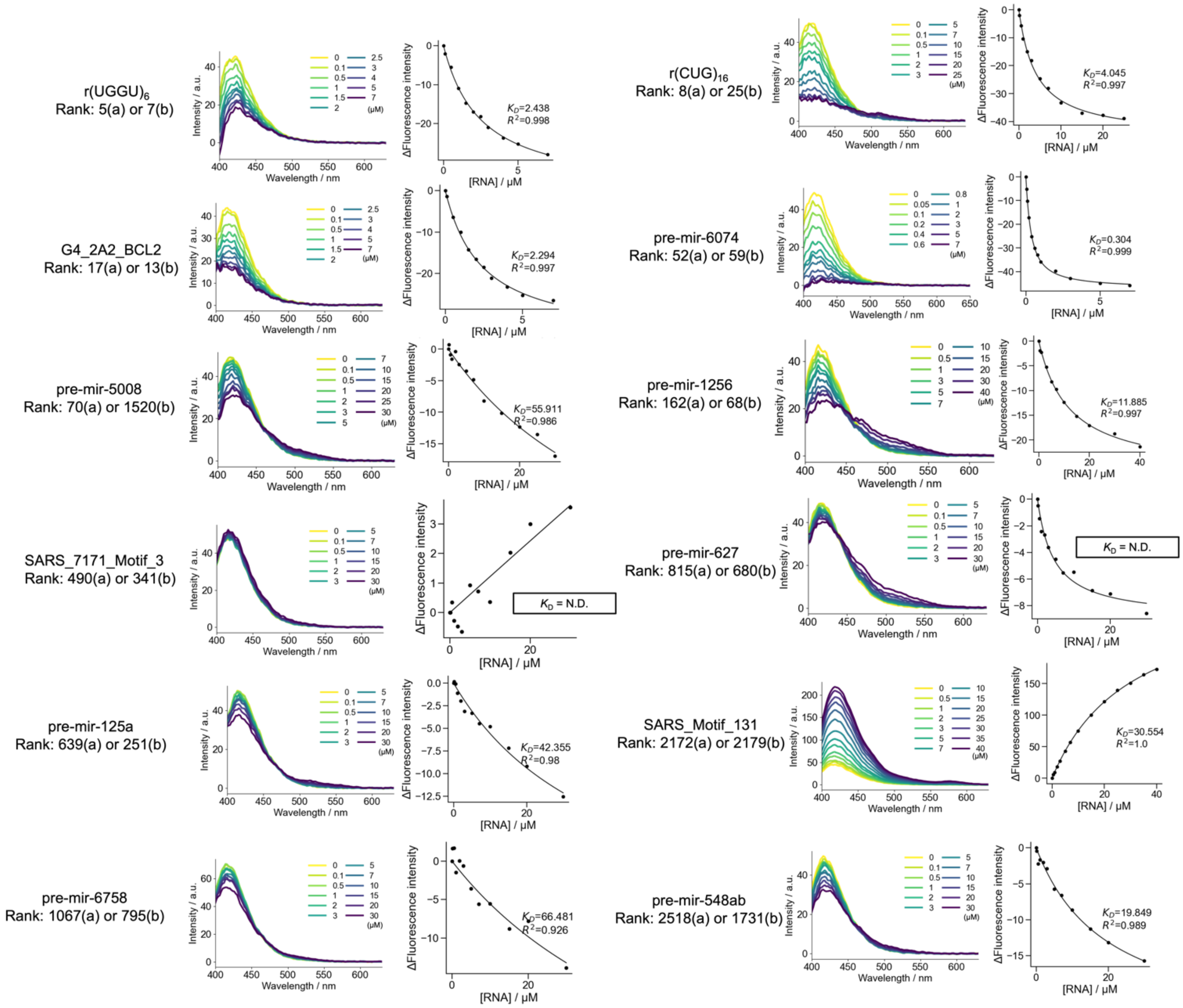

Figure S3. Fluorescence titration experiments to determine the apparent dissociation constants (K_{Dapp}) for the validation of FOREST. Fluorescence titration spectra were measured using amiloride hydrochloride (100 nM) in Fluorescence titration buffer (1% DMSO (v/v), 20 mM phosphate pH 7.0, 20 mM NaCl and 80 mM KCl), upon addition of RNA (0-7 or 0-25, or 0-30, or 0-40 μM) Conditions: λ_{ex}: 381 nm, λ_{em}: 420 nm. Temperature = 20 °C. The representative data and K_{Dapp} values are shown. Two or three independent measurements were performed, and the representative data and K_{Dapp} value are shown.

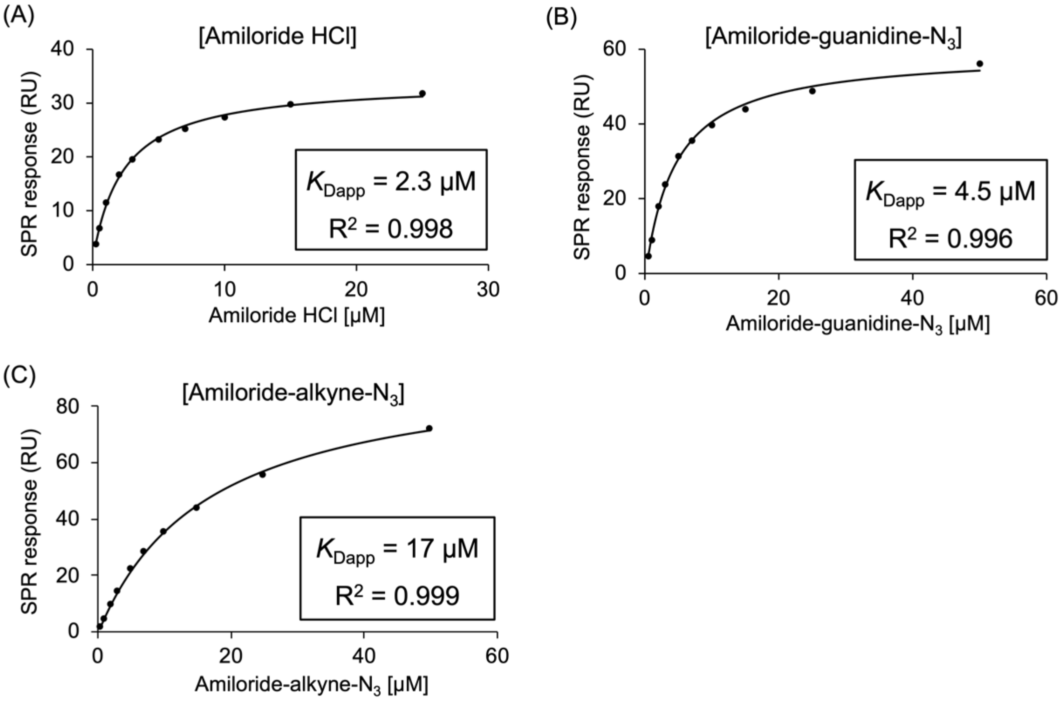

Figure S4. K_{Dapp} calculations of the SPR data with (A) amiloride hydrochloride, (B) Amiloride-guanidine-N_3, and (C) Amiloride-alkyne-N_3. The black dots represent the SPR response, and the black line is the fitting curve obtained by Biacore T200 evaluation software using the steady state affinity model. Three technical replicates were conducted, and the representative data and K_{Dapp} values are shown.

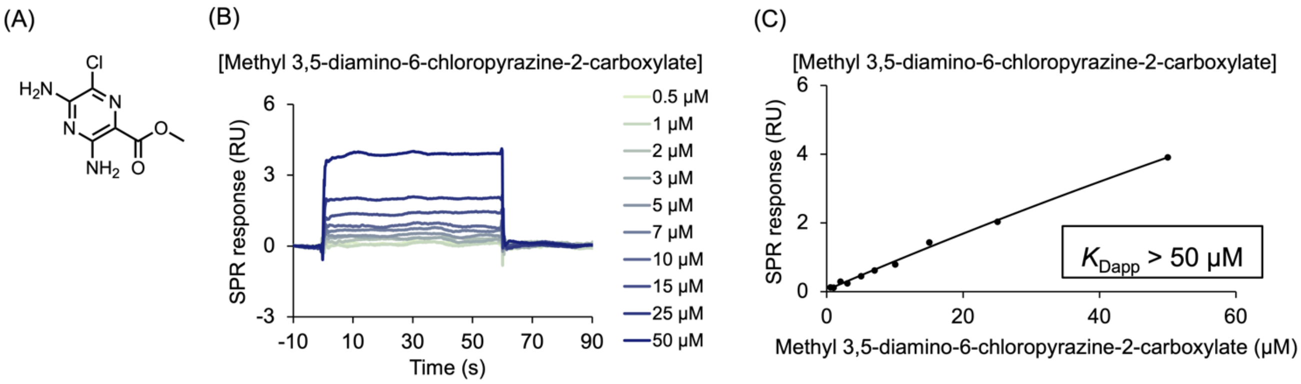

Figure S5. Surface Plasmon Resonance (SPR) analysis of methyl 3,5-diamino-6-chloropyrazine-2-carboxylate. (A) Chemical structure of methyl 3,5-diamino-6-chloropyrazine-2-carboxylate. (B) SPR sensorgram of methyl 3,5-diamino-6-chloropyrazine-2-carboxylate (0-50 μM) with 5′-biotinylated pre-miR-6074 and (C) calculation of the K_{Dapp} value by steady state affinity model. The black dots represent the SPR response, and the black line is the fitting curve. Two technical replicates were performed, and the representative data and K_{Dapp} value are shown.

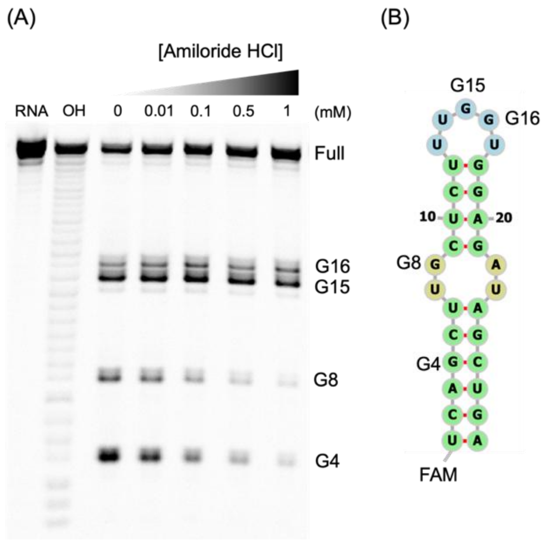

Figure S6. Investigation of the binding site of amiloride on pre-miR-6074 by RNase footprinting assay with RNase T1. (A) Gel image of the inhibition of RNase T1 cleavage by amiloride hydrochloride. Footprinting assay conditions: pre-miR-6074-FAM (1 μM), amiloride hydrochloride (0–1 mM), RNase T1 (270 ng/mL). Buffer: 1% DMSO, 20 mM phosphate pH 7.0, 80 mM KCl, 20 mM NaCl, Reaction time: 15 min at 37 °C. Lanes: pre-miR-6074 only (RNA), alkaline hydrolysis ladder (OH). The electrophoresis was performed on a 16% denaturing polyacrylamide gel containing 20% formamide. (B) Secondary structure of 5'FAM labeled pre-miR-6074 predicted by RNAfold.

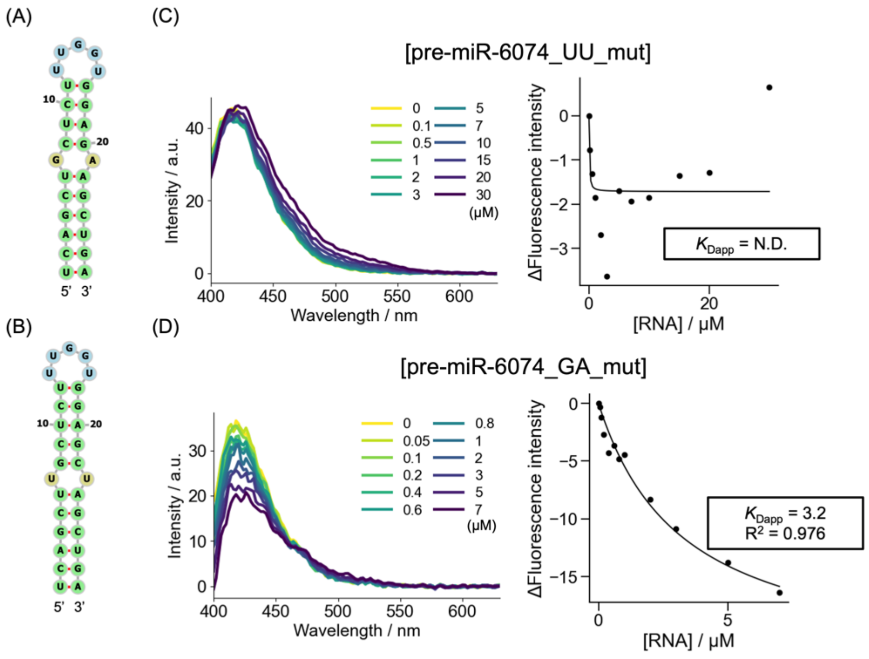

Figure S7. Determination of the binding motif of amiloride on pre-miR-6074 by fluorescence titration with a mutant. (A) Secondary structure of pre-miR-6074_UU_mut and (B) pre-miR-6074_GA_mut predicted by RNAfold. Fluorescence titration experiments to determine the apparent dissociation constant (K_{Dapp}) between amiloride and (C) pre-miR-6074_UU_mut and (D) pre-miR-6074_GA_mut. Fluorescence titration spectra were measured using amiloride hydrochloride (100 nM) in Fluorescence titration buffer (1% DMSO (v/v), 20 mM phosphate pH 7.0, 20 mM NaCl and 80 mM KCl) upon addition of RNA (0-30 or 0-7 μM). Conditions: λ_{ex}: 381 nm, λ_{em}: 420 nm. Temperature = 20 °C. Two or three independent measurements were performed, and the representative data and K_{Dapp} value are shown.

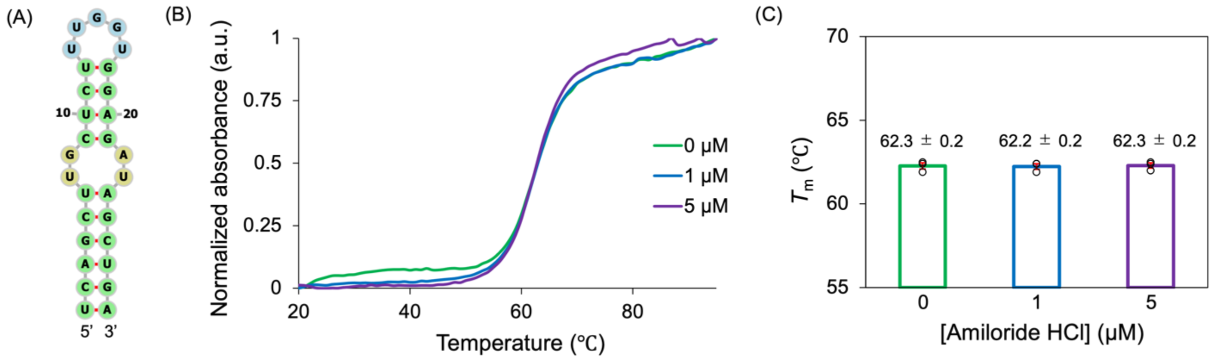

Figure S8. T_m analysis of pre-miR-6074 with amiloride. (A) Secondary structure of pre-miR-6074 predicted by RNAfold. (B) Representative normalized T_m curves of pre-miR-6074 (1 μM) with amiloride hydrochloride (0, 1, and 5 μM) in 1x T_m buffer (1% DMSO, 20 mM phosphate pH 7.0, 20 mM NaCl, and 80 mM KCl). (C) Bar graphs of T_m values (°C). The white circles represent the measured T_m values and the red error bars represent the standard error from three independent measurements. The number on the bar graphs are the T_m values, and they are reported as mean ± standard error.

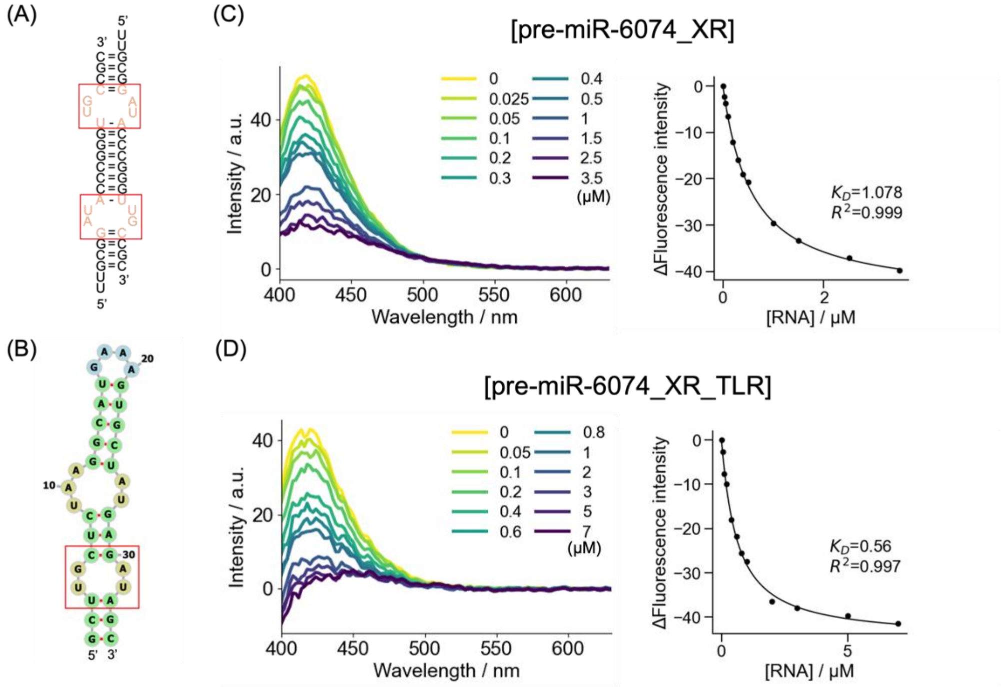

Figure S9. Determination of the binding affinity between amiloride and RNAs used for X-ray crystallographic analysis. Secondary structures of (A) pre-miR-6074_ XR predicted by RNAcofold and (B) pre-miR-6074_ XR_TLR predicted by RNAfold. The red boxes indicate the extracted internal loop motif from pre-miR-6074 for the structural studies. Fluorescence titration experiment to determine the apparent dissociation constant (K_{Dapp}) between amiloride and (C) pre-miR-6074_XR and (D) pre-miR-6074_XR_TLR. Fluorescence titration spectra were measured using amiloride hydrochloride (100 nM) in Fluorescence titration buffer (1% DMSO (v/v), 20 mM phosphate pH 7.0, 20 mM NaCl and 80 mM KCl) upon addition of RNA (0-3.5 or 0-7 μM). Conditions: λ_{ex}: 381 nm, λ_{em}: 420 nm. Temperature = 20 °C. Three independent measurements were performed, and the representative data and K_{Dapp} value are shown.

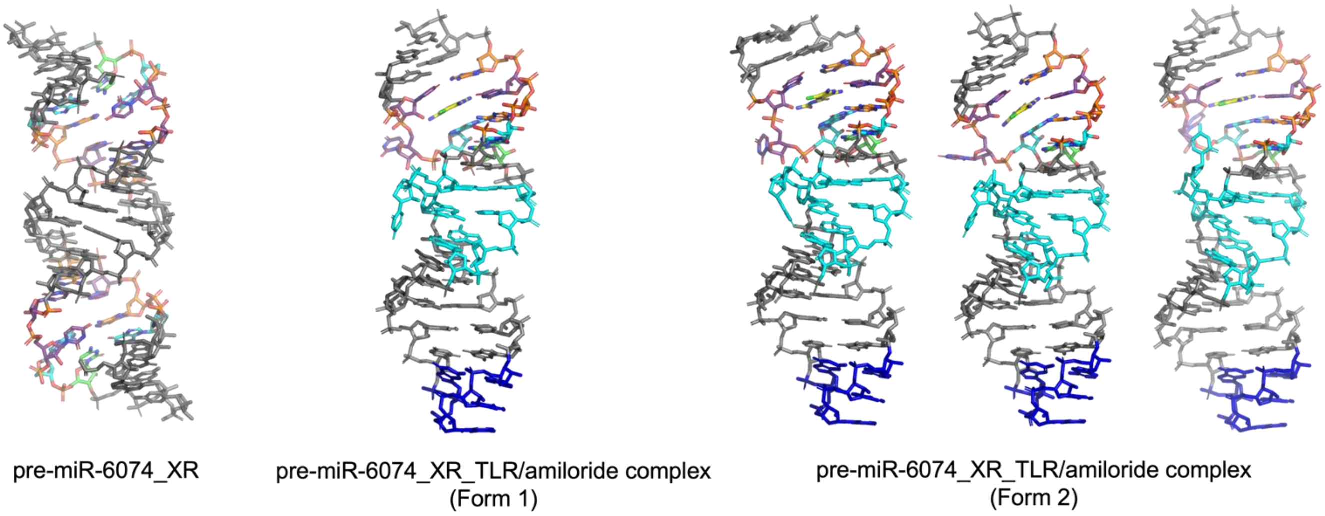

Figure S10. Molecular structures observed in the asymmetric units of the crystals. In the pre-miR-6074_XR_TLR molecule, the tetraloop and tetraloop receptor are colored in blue and cyan, respectively.

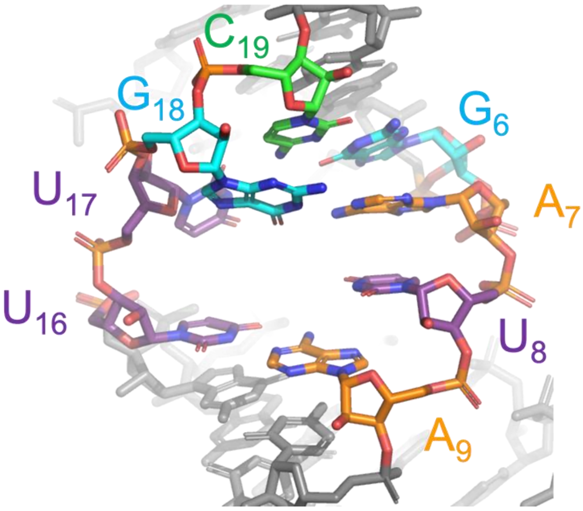

Figure S11. Close-up view of the extracted internal loop motif in the crystal structure of pre-miR-6074_XR.

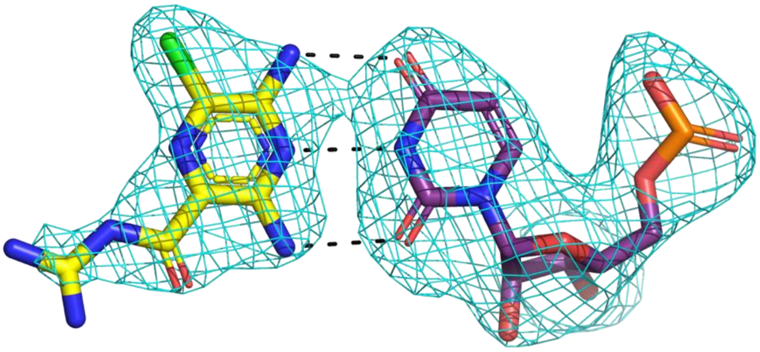

Figure S12. Complementary hydrogen-bonding pattern between amiloride and U (pre-miR-6074_XR_TLR/amiloride complex form 1). The $2mF_o$-DF_c electron density is contoured at a 1.5 σ level. Hydrogen bonds are illustrated with dashed lines.

Synthesis of Amiloride-guanidine-N_3

Synthesis scheme was developed based on a previous report(6)

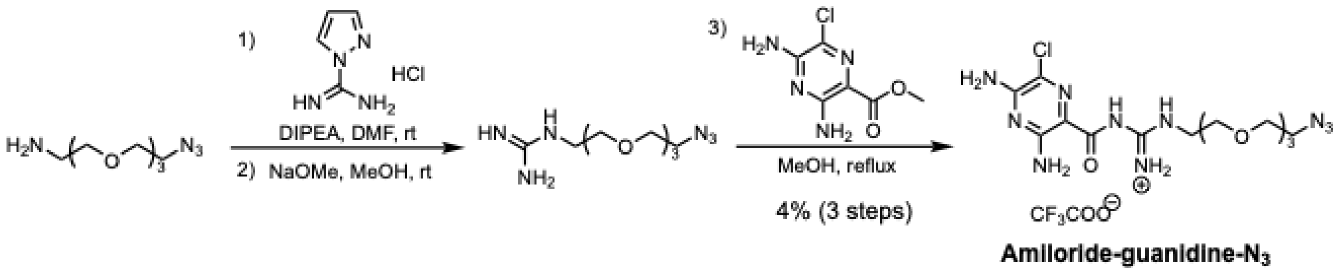

Scheme S1 Synthesis of Amiloride-guanidine-N_3

Under an argon atmosphere, 11-azido-3,6,9-trioxaundecan-1-amine (49.6 mL, 0.250 mmol) and 1-amidinopyrazole hydrochloride (51.3 mg, 0.350 mmol) were dissolved in 1.6 mL of DMF/DIPEA (3:1) solution. The mixture was stirred at room temperature for 16 h, then diluted with diethylether (10 mL). The cloudy liquid was decanted, and the residual yellow oil was washed with diethylether (10 mL x2). The oil was dried *in vacuo*, then dissolved in MeOH (0.5 mL). NaOMe (27.0 mg, 0.500 mmol) was added to the MeOH solution, and the mixture was stirred at room temperature for 30 min. The mixture was then filtered, dried, and redissolved in MeOH (0.5 mL). To the MeOH solution, methyl 3,5-diamino-6-chloropyrazine-2-carboxylate (20.2 mg, 0.100 mmol) was added, and the reaction mixture was let refluxed for 25 h. The reaction was then concentrated *in vacuo*, dissolved in DMSO, filtrated, and purified by reversed-phase HPLC to afford **Amiloride-guanidine-N_3** as a yellow oil (4%, over 3 steps). The yield was determined by quantitative ^{1}H-NMR measurement using maleic acid as an internal standard. HPLC conditions; A: 0.1% TFA in distilled water, B: 0.1% TFA in MeCN; B: 5% → 60% (∼20 min) → 100% (∼25 min) → 100%(∼30 min) → 5% (~31 min). Flow rate = 4 mL/min; Temp. = 35 °C; UV = 254 nm, C-18 column (Nacalai Tesque: COSMOSIL 5C_{18}-AR-II, 10 × 250 mm). ^{1}H NMR (500 MHz, DMSO-d_6) δ (ppm) 10.59 (1H, s), 9.30 (1H, s), 8.93 (1H, s), 8.74 (1H, s), 7.93 (1H, br), 7.43 (3H, brs), 3.62-3.34 (16H).* *Signal overlaps with the water signal from the solvent. ^{13}C NMR (125 MHz, DMSO-d_6) δ (ppm) 165.4, 156.0, 154.3, 153.7, 119.71, 109.1, 69.8, 69.8, 69.8, 69.7, 69.2, 67.7, 50.0, 41.1. ESI-HRMS (m/z): [M-CF_3COO]$^+$calcd for $C_{14}H_{24}ClN_{10}O_4^+$ 431.1666, found 431.1679.

Synthesis of Amiloride-alkyne-N_3

Synthesis scheme was developed based on previous reports(7–10)

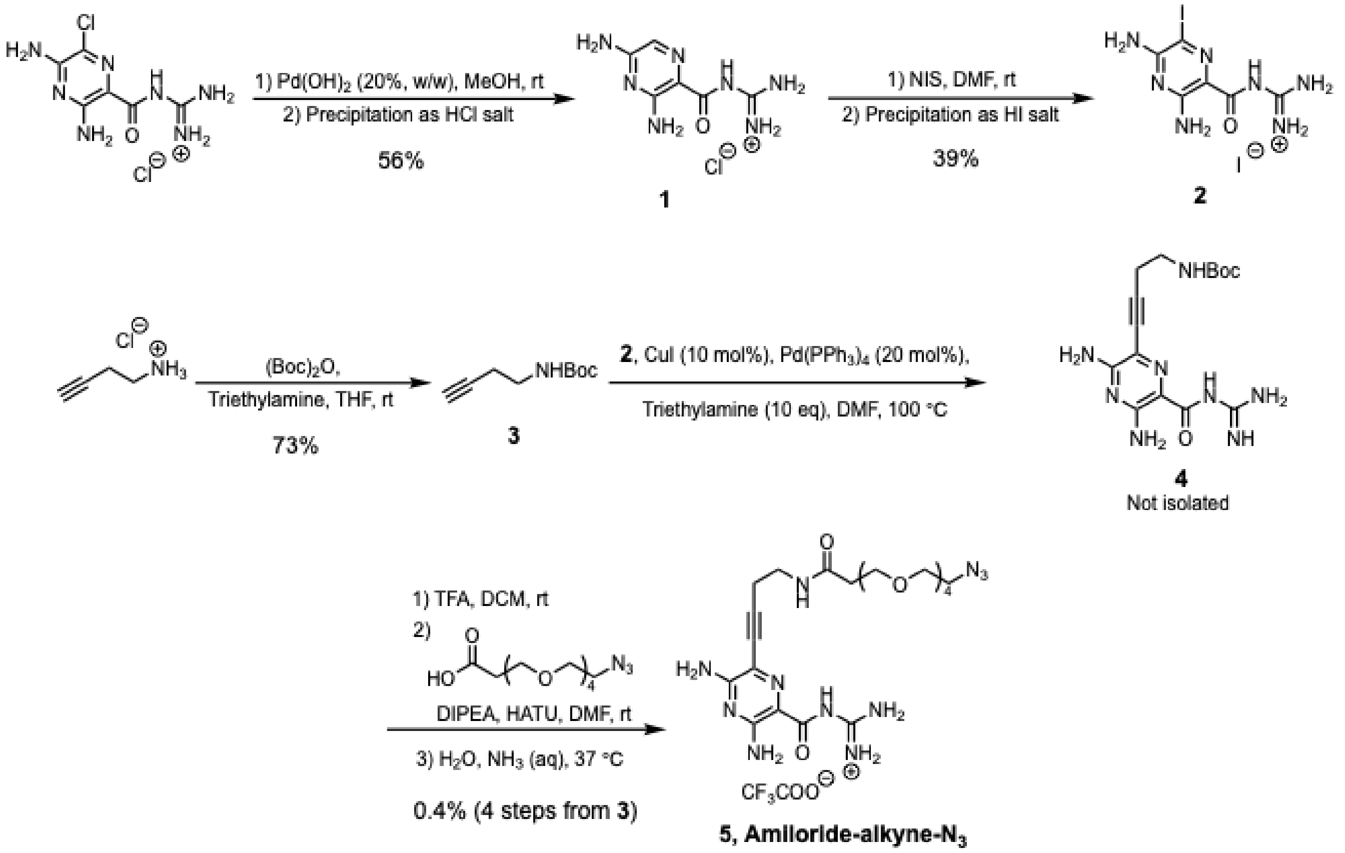

Scheme S2 Synthesis of Amiloride-alkyne-N_3

Synthesis of compound 1

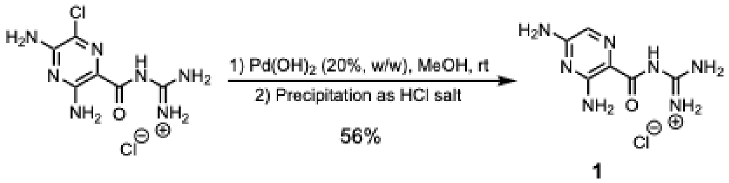

To a flask which amiloride hydrochloride (1000 mg, 3.76 mmol) was dissolved in MeOH (50 mL), $Pd(OH)_2$ on activated carbon (200 mg) was added. The reaction flask was then charged with H_2 (g) and the solution was stirred at room temperature for 23 hours. After filtering the reaction mixture through a celite cake, solvent was then removed *in vacuo*. The crude was then dissolved in MeOH, and the solution was acidified by the addition of 6M HCl. Formation of white/yellow precipitate was observed while diethylether was added to the methanol solution, where the precipitate was collected and dried afford hydrochloride salt of **1** as a yellow solid (498 mg, 56%).

^{1}H NMR* (600 MHz, DMSO-d_6) δ (ppm) 10.94 (1H, s), 8.57 (2H, brs), 8.47 (2H, brs), 7.48 (3H, br),** 7.33 (1H, s).** *One N-H signal is not observed. ** Signals are overlapping.^{13}C NMR (151 MHz, DMSO-d_6) δ (ppm) 166.1, 157.5, 156.6, 155.2, 122.6, 109.7. ESI-HRMS (*m/z*): $[M\text{-}Cl]^+$ calcd for $C_6H_{10}N_7O^+$ 196.0942, found: 196.0949

Synthesis of compound 2

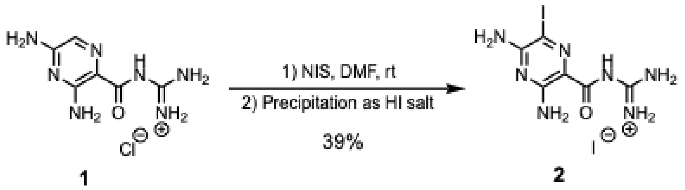

Under an argon atmosphere, **1** (200 mg, 0.863 mmol) and *N*-Iodosuccinimide (485 mg, 2.16 mmol) was dissolved in DMF (15 mL). After stirring the reaction 26.5 hours, the reaction was quenched by the addition of sat. NaS_2O_3 solution. After the removal of the solvent *in vacuo*, the crude was then resuspended in water (10 mL). Subsequently, the solid was collected by filtration, and washed with water (10 mL, x2) and acetone (10 mL, x2). The collected solid was then redissolved in MeOH and 57% Hydriodic acid was added to the solution. Formation of brown precipitate was observed while diethylether was added to the methanol solution, where the precipitate was collected and dried to afford the hydroiodide salt of **2** as a brown solid (149 mg, 39%). ^{1}H NMR (600 MHz, DMSO-*d*$_6$) δ (ppm) 10.49 (1H, s), 8.60 (2H, b), 8.16 (2H, brs), 7.35 (4H, br). ^{13}C NMR (151 MHz, DMSO-*d*$_6$) δ (ppm) 165.3, 157.1, 155.7, 154.8, 113.2, 88.1. ESI-HRMS (*m/z*): $[M-I]^+$ calcd for $C_6H_9IN_7O^+$ 321.9908, found: 321.9946.

Synthesis of compound 3

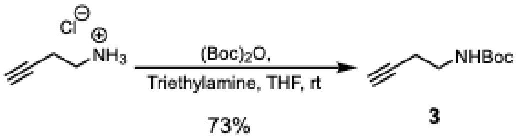

Under an argon atmosphere, 3-butyn-1-amine hydrochloride (50.0 mg, 0.474 mmol), di-*tert*-butyl-dicarbonate (109 µL, 0.474 mmol), and triethylamine (132 µL, 0.947 mmol) were dissolved in THF (2.4 mL). After stirring the reaction 17 hours, the reaction mixture was diluted with DCM (7 mL) and H_2O (7 mL). The aqueous phase was further extracted with DCM (7 mL, x3), and the combined organic phase was dried over $MgSO_4$, concentrated. The crude was purified by silica gel column chromatography (ethyl acetate: hexane 9:1) to afford **3** as a colorless oil (58.4 mg, 73%).

^{1}H NMR (400 MHz, $CDCl_3$) δ (ppm) 4.85 (1H, br), 3.28 (2H, q, *J* = 6.0 Hz), 2.38 (2H, td, *J* = 6.4 and 2.4 Hz), 1.99 (1H, t, *J* = 2.4 Hz), 1.44 (9H). ESI-HRMS (*m/z*): $[M+Na]^+$ calcd for $C_9H_{15}NNaO_2^+$ 192.0995, found: 192.0993.
The ^{1}H NMR of **3** is consistent with previous reports.(9,11)

Synthesis of compound 4

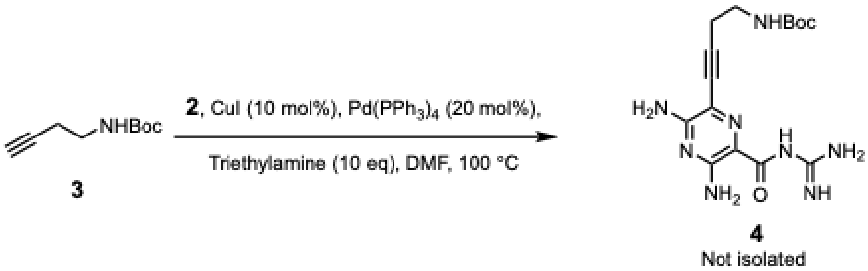

Under an argon atmosphere, **2** (21.5 mg, 0.0601 mmol), **3** (30.5 mg, 0.180 mmol), and triethylamine (84 µL, 0.62 mmol) were dissolved in DMF (1.2 mL). After bubbling the solution with argon for 30 minutes, CuI (1.1 mg, 0.0058 mmol) and $Pd(PPh_3)_4$ (6.9 mg, 0.0060 mmol) were added to the reaction mixture, then the solution was heated at 100 ℃ for total of 14 hours. The solution was cooled down to room temperature, and additional $Pd(PPh_3)_4$ (6.9 mg,

0.0060 mmol) was added to the reaction mixture, which was heated 100 ℃ for another 12 hours. After cooling down the mixture to room temperature, it was filtered through celite cake and concentrated under vacuum. The crude was then purified using amino silica gel column chromatography (DCM: MeOH = DCM only → 19:1 → 9:1 → 6:1 → 3:1) to afford a brown solid mixture containing **4** (1.9 mg).

Synthesis of compound 5 (Amiloride-alkyne-N_3)

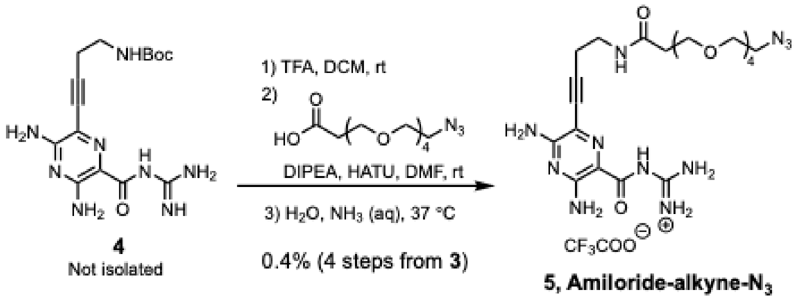

The brown solid mixture containing **4** (1.9 mg) was dissolved in TFA (250 μL) and DCM (250 μL), and the solution was stirred at room temperature for 1 hour. After the removal of the solvent *in vacuo* and coevaporation with MeCN (x5), DIPEA (4.6 μL, 26 μmol) and DMF (100 μL) was added to the reaction flask. To the flask, azido-PEG4-acid (1.7 mg, 5.8 μmol) in DMF (150 μL) and HATU (3.0 mg, 7.9 μmol) was added, and the mixture was stirred at room temperature for 5 hours. The reaction was then concentrated *in vacuo*, dissolved in DMSO, filtrated, and the crude was collected using reversed-phase HPLC.

HPLC conditions; A: 0.1% TFA in distilled water, B: 0.1% TFA in MeCN; B: 5% → 50% (~20 min) → 100% (~25 min) → 100% (~30 min) → 5% (~31 min). HPLC conditions; A: 0.1% TFA in distilled water, B: 0.1% TFA in MeCN; B: 5% → 50% (∼20 min) → 100% (∼25 min) → 100%(∼30 min) → 5% (~31 min). Flow rate = 4 mL/min; Temp. = 35 °C; UV = 254 nm, C-18 column (Nacalai Tesque: COSMOSIL $5C_{18}$-AR-II, 10 × 250 mm).

After the removal of the solvent under a lyophilizer, the crude was redissolved in 14% NH_3 aqueous solution (0.6 mL). The mixture was incubated at 37 ℃ for 1 hour. After the removal of ammonia under vacuum, 0.1% TFA aqueous solution (1 mL) was added, purified using reversed-phase HPLC twice to afford **5** as yellow solid (0.4%). The yield was determined by quantitative NMR using maleic acid as an internal standard.

HPLC conditions; A: 0.1% TFA in distilled water, B: 0.1% TFA in MeCN; B: 5% → 50% (~20 min) → 100% (~25 min) → 100% (~30 min) → 5% (~31 min). HPLC conditions; A: 0.1% TFA in distilled water, B: 0.1% TFA in MeCN; B: 5% → 40% (∼20 min) → 100% (∼25 min) → 100%(∼30 min) → 5% (~31 min). Flow rate = 4 mL/min; Temp. = 35 °C; UV = 254 nm, C-18 column (Nacalai Tesque: COSMOSIL $5C_{18}$-AR-II, 10 × 250 mm).

^{1}H NMR (600 MHz, DMSO-d_6) δ (ppm) 10.70 (1H, s), 8.59 (2H, s), 8.17-8.14 (3H, m)*, 7.79 (1H, brs), 7.50-7.47 (2H, m)*, 6.99-6.93 (1H, m), 3.59-3.33 (20H)**, 2.59 (2H, br), 2.33 (2H, br).*Signals are overlapping. **Signal overlaps with the water signal from the solvent.^{13}C NMR (151 MHz, DMSO-d_6) δ (ppm) 170.7, 165.5, 157.8, 155.5, 154.8, 114.2, 110.7, 93.2, 76.2, 69.8, 69.8, 69.8, 69.7, 69.6, 69.6, 69.5, 69.3, 66.7, 50.0, 37.3, 36.3, 20.4. ESI-HRMS (*m/z*): $[M-CF_3COO]^+$ calcd for $C_{21}H_{34}ClN_{11}O_6^+$ 536.2689, found 536.2691.

Amiloride-guanidine-N$_3$ ^{1}H NMR (500 MHz, DMSO-d_6)

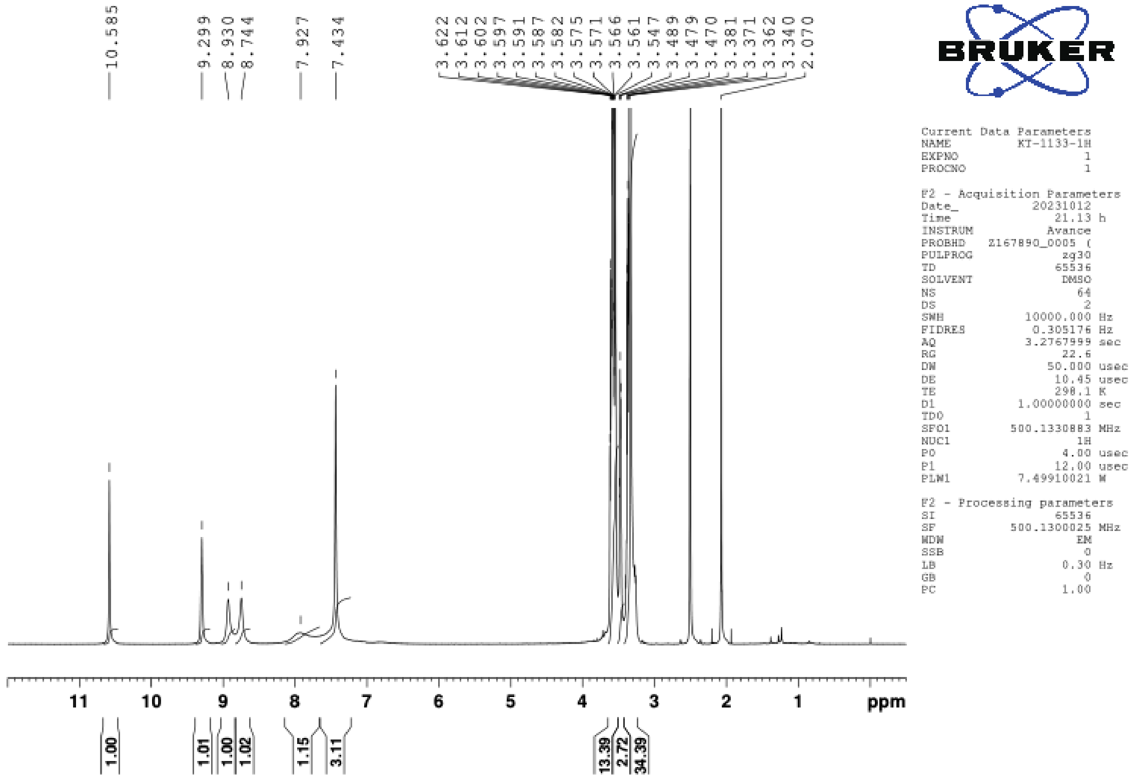

Amiloride-guanidine-N$_3$ ^{13}C NMR (126 MHz, DMSO-d_6)

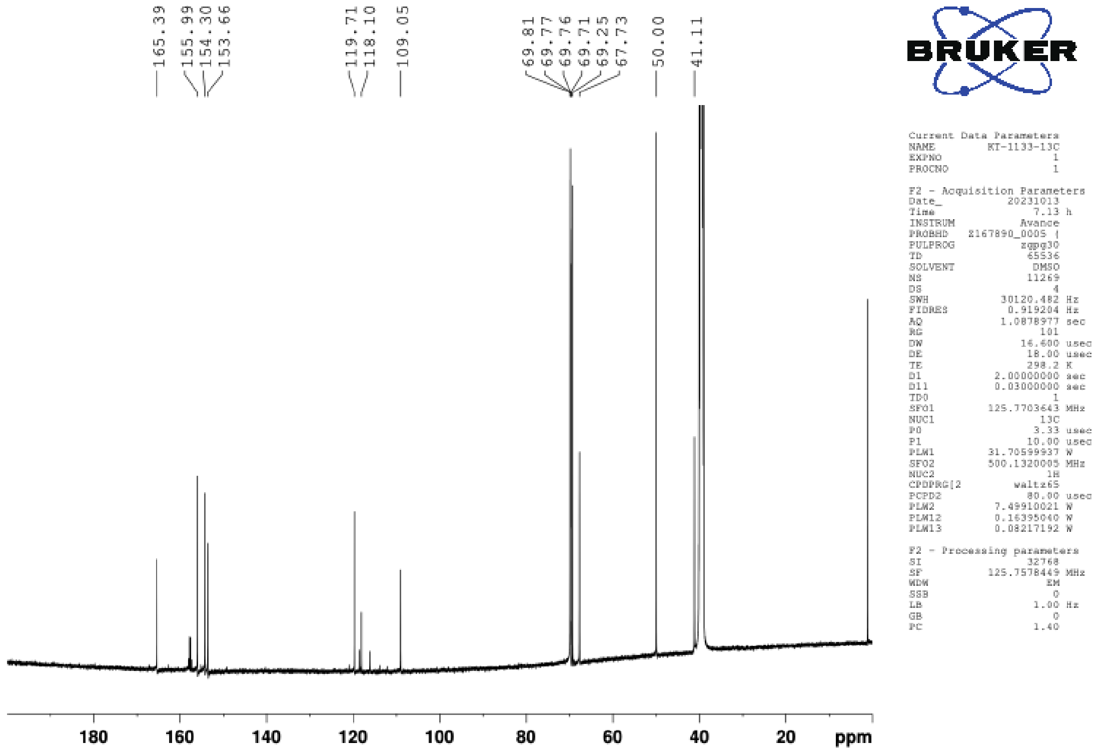

1 ^{1}H NMR (600 MHz, DMSO-d_6)

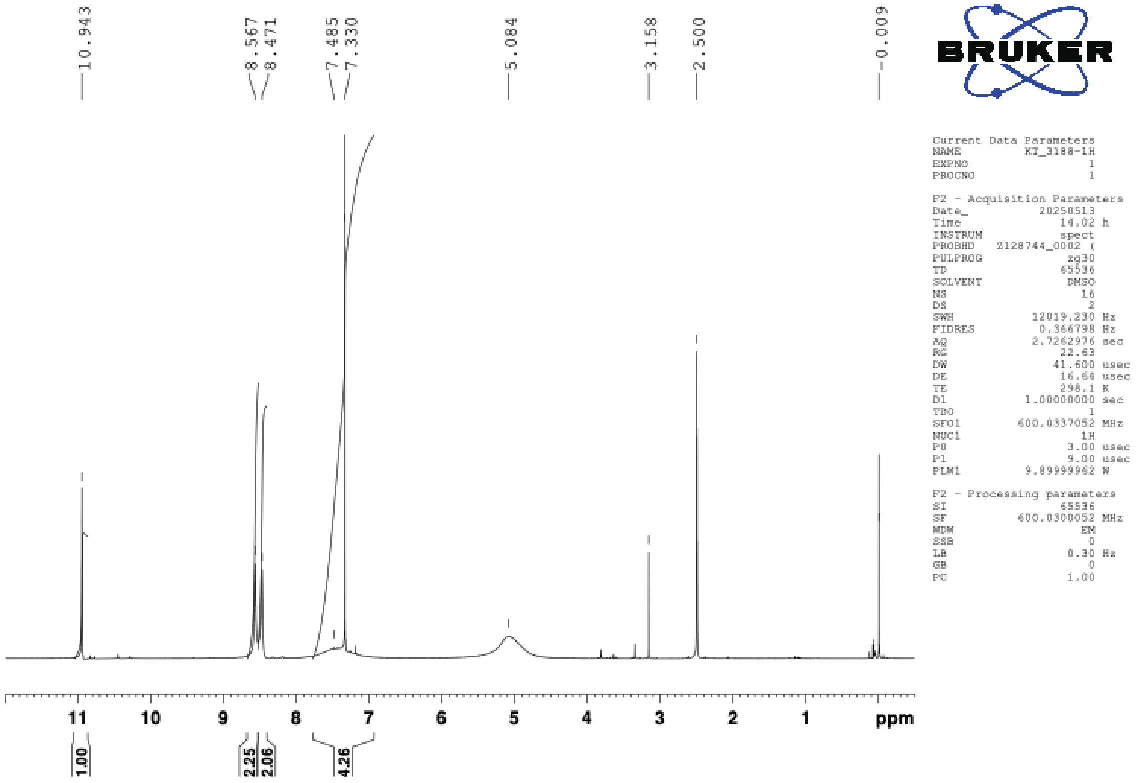

1 ^{13}C NMR (151 MHz, DMSO-d_6)

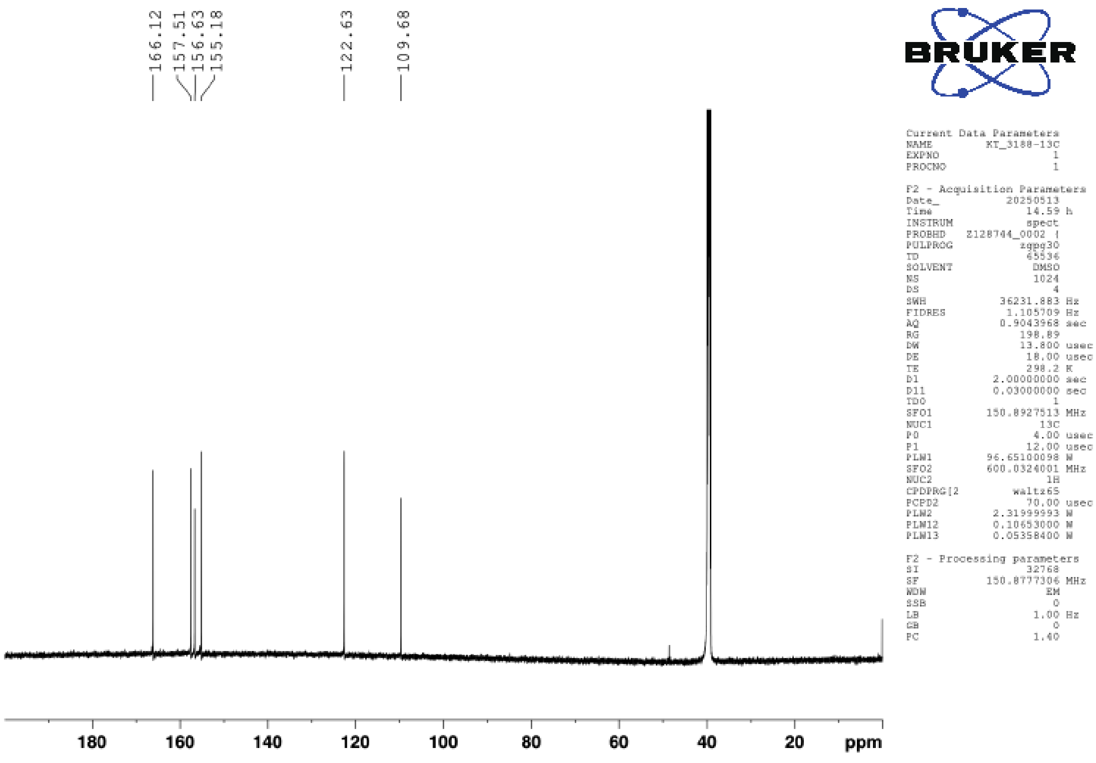

2 ^{1}H NMR (600 MHz, DMSO-d_6)

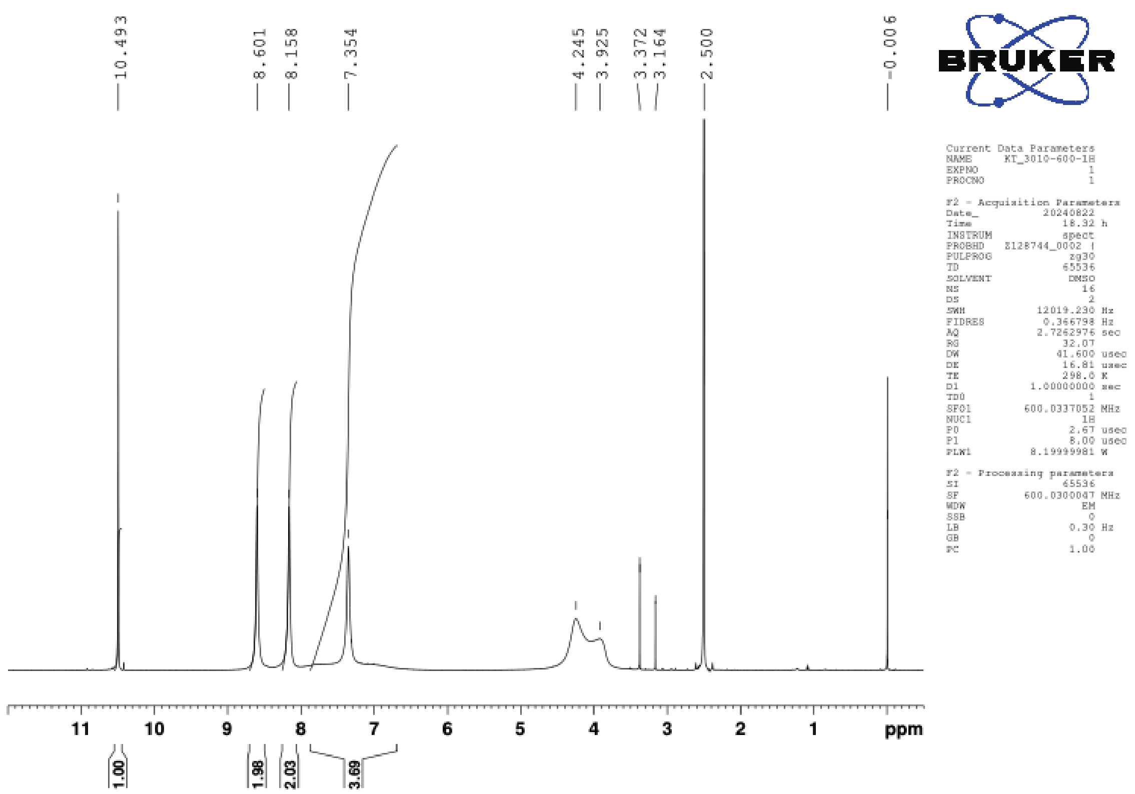

2 ^{13}C NMR (151 MHz, DMSO-d_6)

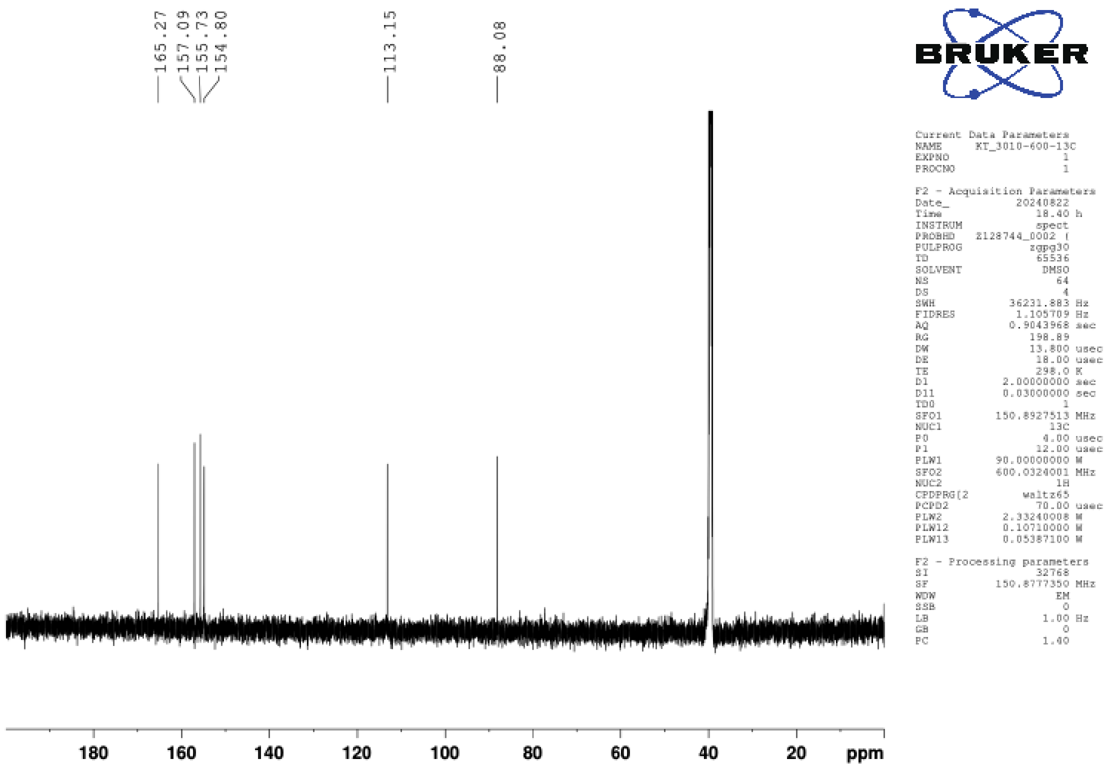

3 ^{1}H NMR (400 MHz, $CDCl_3$)

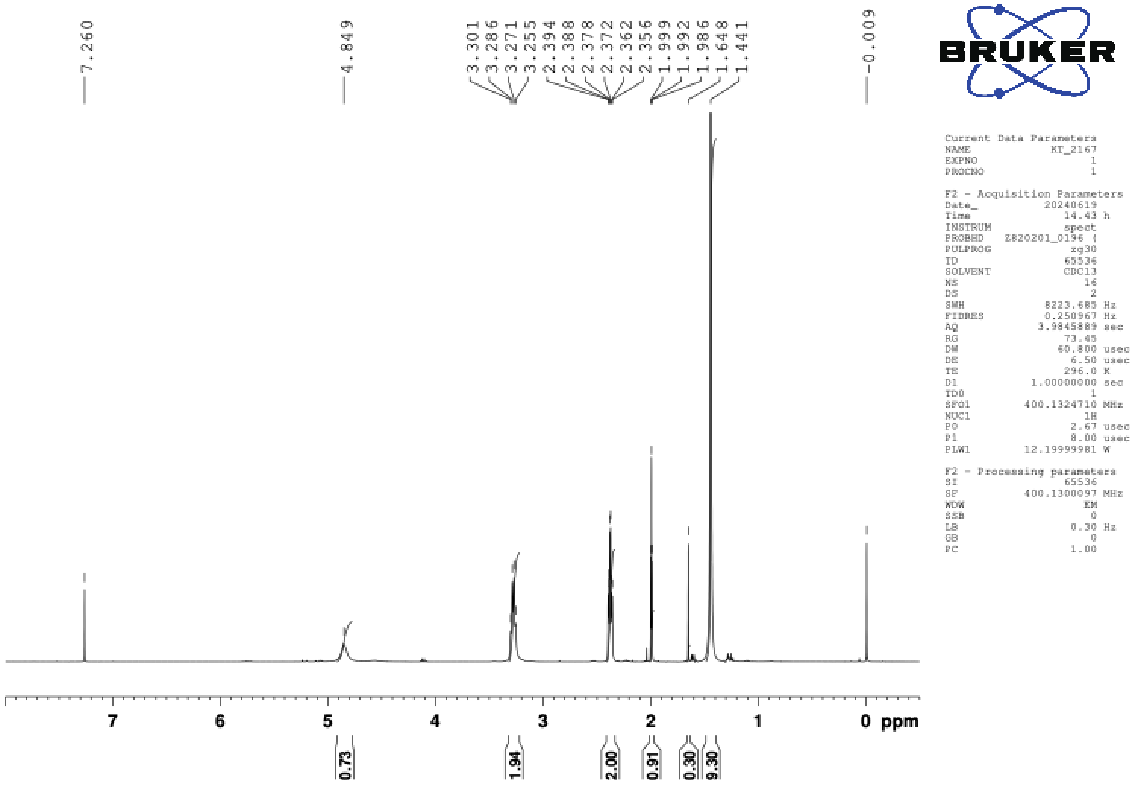

Amiloride-alkyne-N_3 ^{1}H NMR (600 MHz, DMSO-d_6)

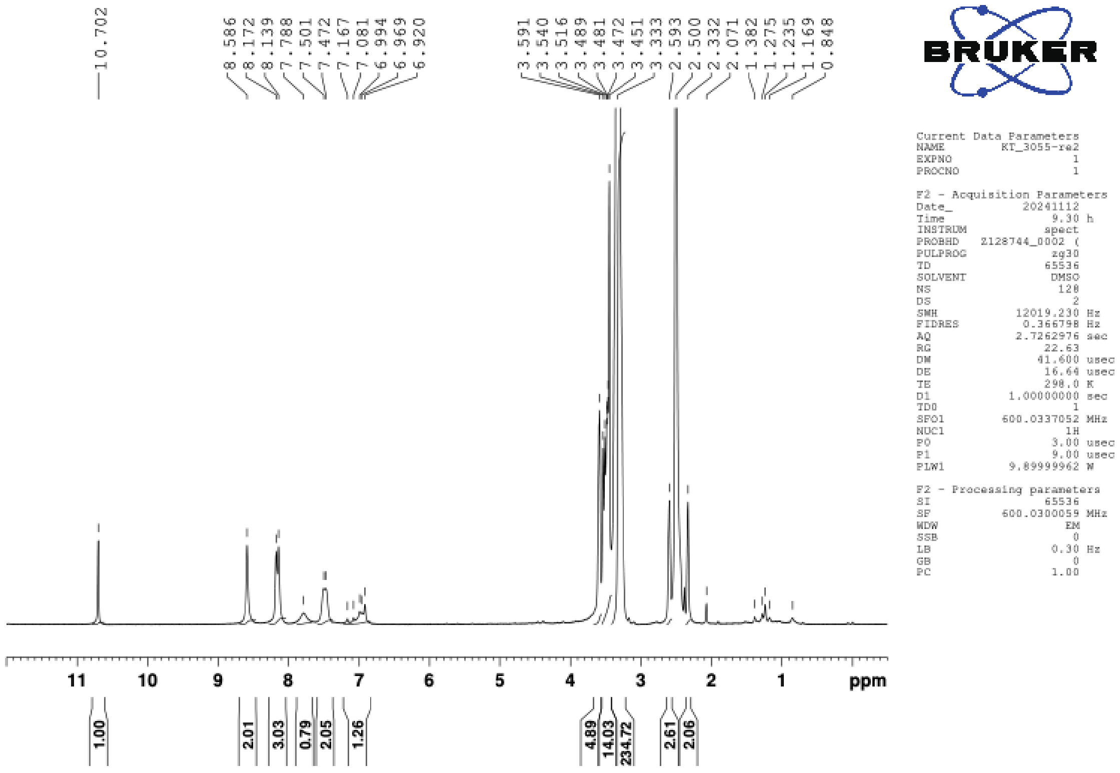

Amiloride-alkyne-N$_3$ ^{13}C NMR (151 MHz, DMSO-d_6)

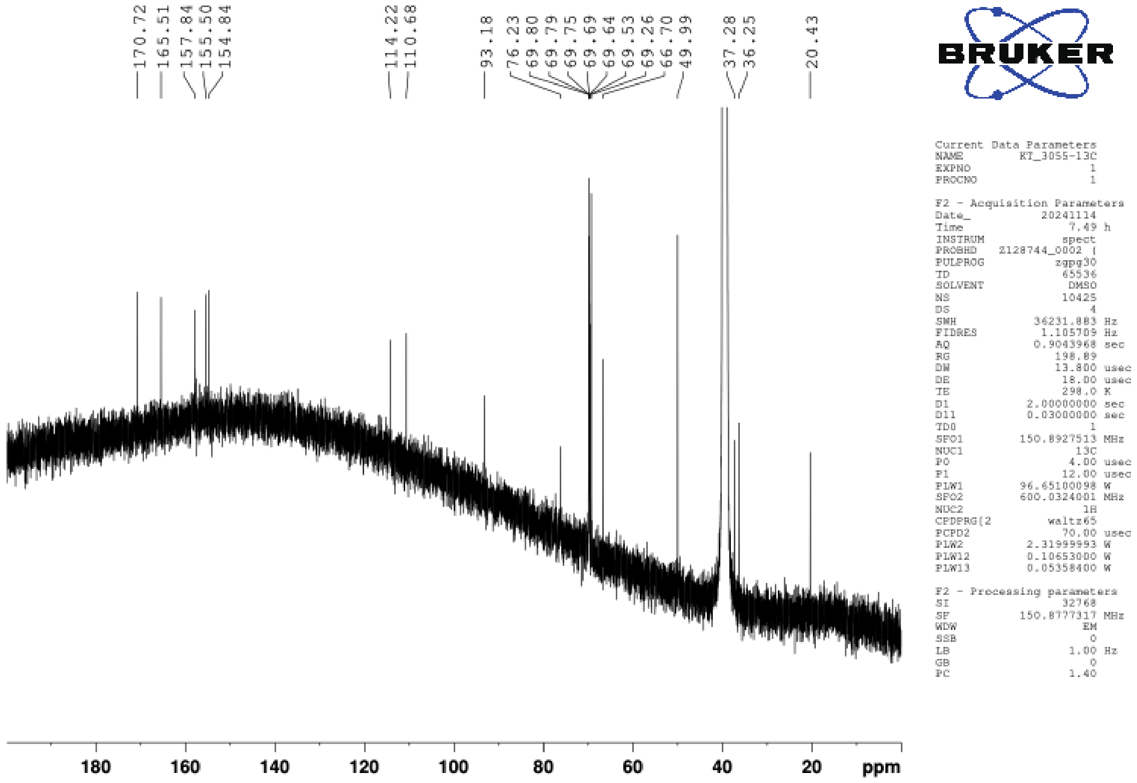

SI Reference

1. R. Nagasawa, K. Onizuka, K. R. Komatsu, E. Miyashita, H. Murase, K. Ojima, S. Ishikawa, M. Ozawa, H. Saito and F. Nagatsugi, *Commun. Chem.*, 2024, **7**, 98.
2. R. Nagasawa, K. Onizuka, R. Iwata, K. Tsuzuki, K. R. Komatsu, E. Miyashita, S. Dantsuji, H. Murase, H. Saito and F. Nagatsugi, *RSC Chem. Biol.*, 2026, **7**, 49–57.
3. R. Lorenz, S. H. Bernhart, C. Höner zu Siederdissen, H. Tafer, C. Flamm, P. F. Stadler and I. L. Hofacker, *Algorithms Mol. Biol.*, 2011, **6**, 26.
4. P. Kerpedjiev, S. Hammer and I. L. Hofacker, *Bioinformatics*, 2015, **31**, 3377–3379.
5. F. H. Stootman, D. M. Fisher, A. Rodger and J. R. Aldrich-Wright, *Analyst*, 2006, **131**, 1145–1151.
6. M. Murai, S. Habu, S. Murakami, T. Ito and H. Miyoshi, *Biosci., Biotechnol., Biochem.*, 2015, **79**, 1061–1066.
7. N. N. Patwardhan, L. R. Ganser, G. J. Kapral, C. S. Eubanks, J. Lee, B. Sathyamoorthy, H. M. Al-Hashimi and A. E. Hargrove, *Med. Chem. Comm*, 2017, **8**, 1022–1036.
8. J. Bergare, L. Kingston, D. J. Guly, J. Dolan, R. J. Lewis and C. S. Elmore, *J. Labelled Compd. Radiopharm.*, 2020, **63**, 434–441.
9. L. Adriaenssens, J. L. Acero Sánchez, X. Barril, C. K. O'Sullivan and P. Ballester, *Chem. Sci.*, 2014, **5**, 4210–4215.
10. H. Matthews, M. Ranson, J. D. A. Tyndall and M. J. Kelso, *Bioorg. Med. Chem. Lett.*, 2011, **21**, 6760–6766.
11. M. Li and D. J. Dixon, *Org. Lett.*, 2010, **12**, 3784–3787.
